# Supplementary material for: Experimentally validated memristive memory augmented neural network with efficient hashing and similarity search
Source: Nat Commun. 2022 Oct 21;13:6284. doi: 10.1038/s41467-022-33629-7 (PMC9587027; doi:10.1038/s41467-022-33629-7)
Supplement: Supplementary file 1 — Supplementary Information [file 41467_2022_33629_MOESM1_ESM.pdf]

Supplemental Information

Experimentally validated memristive memory augmented neural network with efficient hashing and similarity search

Ruibin Mao[1], Bo Wen[1], Arman Kazemi[2,3], Yahui Zhao[1], Ann Franchesca Laguna[3,+], Rui Lin[1], Ngai Wong[1], Michael Neimier[3], X. Sharon Hu[3], Xia Sheng[2], Catherine E. Graves[2,*], John Paul Strachan[4,5,*], and Can Li[1,*]

[1]Department of Electrical and Electronic Engineering, The University of Hong Kong, Hong Kong SAR, China
[2]Hewlett Packard Labs, Hewlett Packard Enterprise, Milpitas, CA, USA
[3]Department of Computer Science and Engineering, University of Notre Dame, Notre Dame, IN, USA
[4]Peter Grünberg Institut (PGI-14), Forschungszentrum Jülich GmbH, Jülich, Germany
[5]RWTH Aachen University, Aachen, Germany
[+]Current address: Department of Computer Technology, De La Salle University, Manila, Philippines
[*]canl@hku.hk, j.strachan@fz-juelich.de, catherine.graves@hpe.com

Contents

1 Supplementary Figures **2**

2 Supplementary Tables **16**

3 Supplementary Notes **17**

4 Supplementary References **40**

1 Supplementary Figures

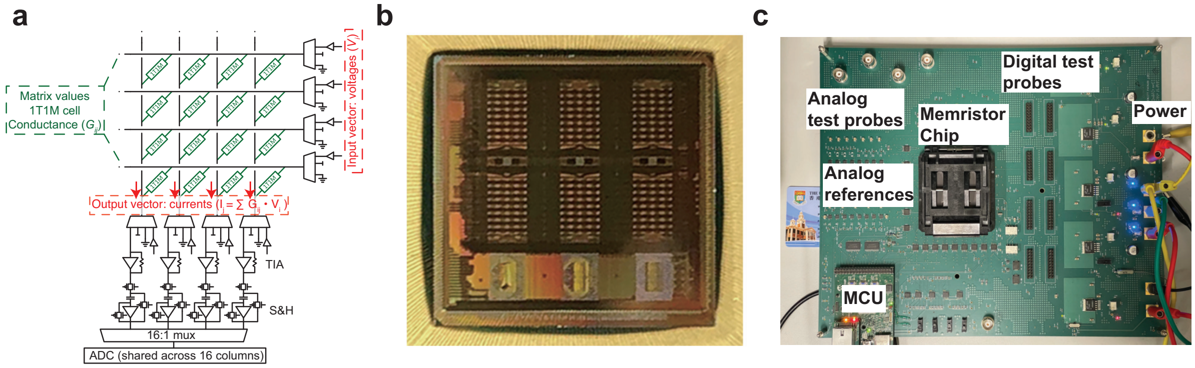

Supplementary Figure 1: CMOS-integrated crossbar test platform and experimental setup. a, Circuit schematic of a crossbar for matrix multiplication with peripheral circuits. It consists of a 1T1R crossbar, row muxes, column muxes, transimpedence amplifiers (TIA), sample and hold (S&H) and ADC. **b**, Picture of a wire-bonded integrated memristor chip. It contains three 64×64 1T1R crossbar arrays. **c**, Measurement board with the integrated memristor chip that connects to a general purpose computer through a microcontroller (MCU).

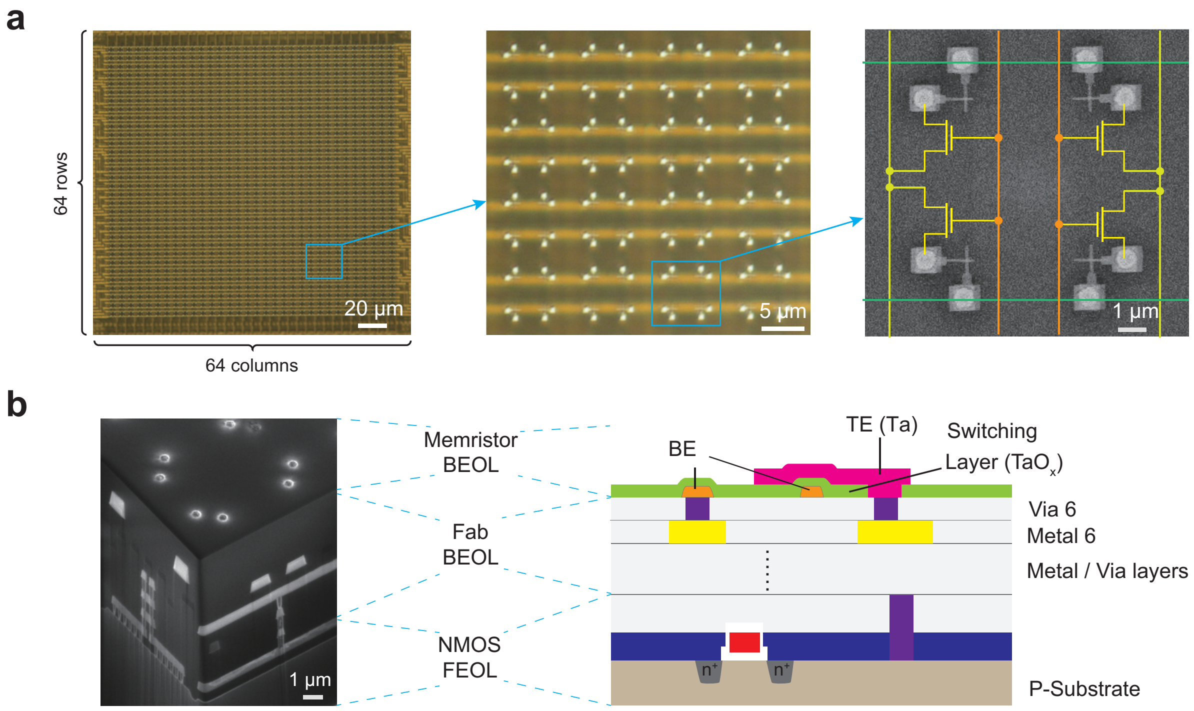

Supplementary Figure 2: Images of our integrated memristive chip. a, Optical top view images of the crossbar arrays. **b**, Cross-section view of the 1T1R structure.

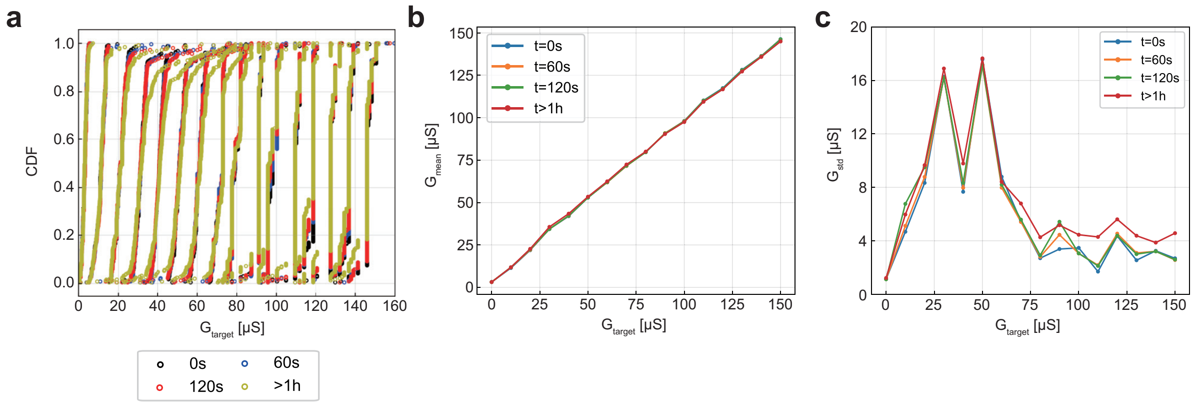

Supplementary Figure 3: Memristor conductance relaxation statistics in a crossbar array. **a**, Cumulative distribution function on 16 distinct programmed conductance states at different time period. We run the test on a 64x64 array where we divide it into 4x4 blocks and program them to 16 conductance states with an iterative write-and-verify programming scheme. **b**, Relation between mean conductance at different time periods and target conductance. **c**, Relation between the standard deviation at different time periods and the target conductance.

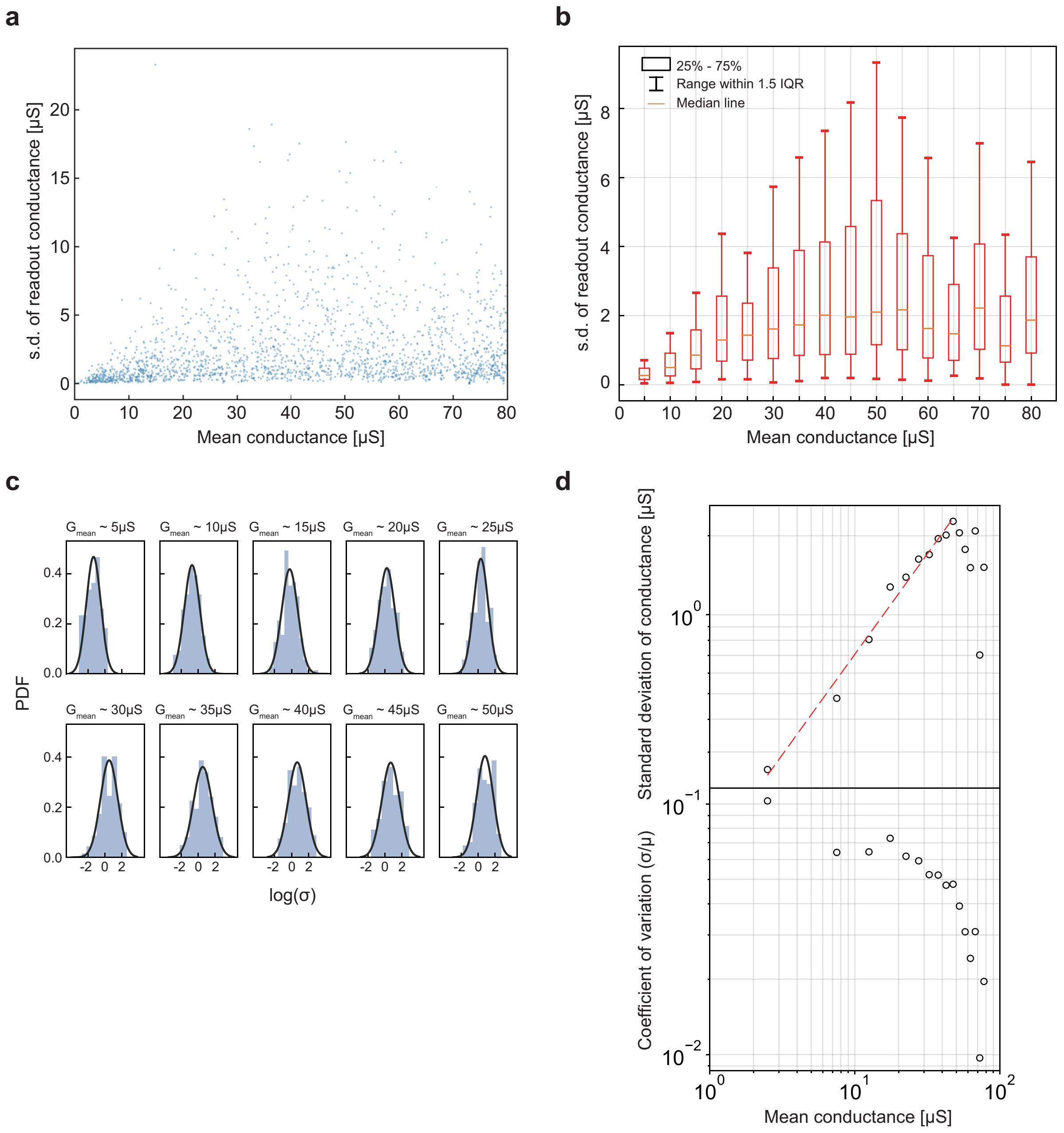

Supplementary Figure 4: Memristor conductance fluctuations in a crossbar array. a, Relation between the standard deviation of 1000 readout conductance and the mean value at different conductance states. We test the whole 64x64 array. **b**, Boxplot of the standard deviation with respect to different conductance states. **c**, Distribution of logarithm of standard deviation at different conductance levels. The distributions show that the device-to-device variation in terms of conductance fluctuation exhibits a lognormal distribution. **d**, The mean value of the logarithm of standard deviation exhibits a linear dependence on the conductance state at the low conductance range ($< 50\,\mu S$) and tends to maintain within a certain range at higher conductance range. The coefficient of the variation brought by the read fluctuation decreases as the conductance increases.

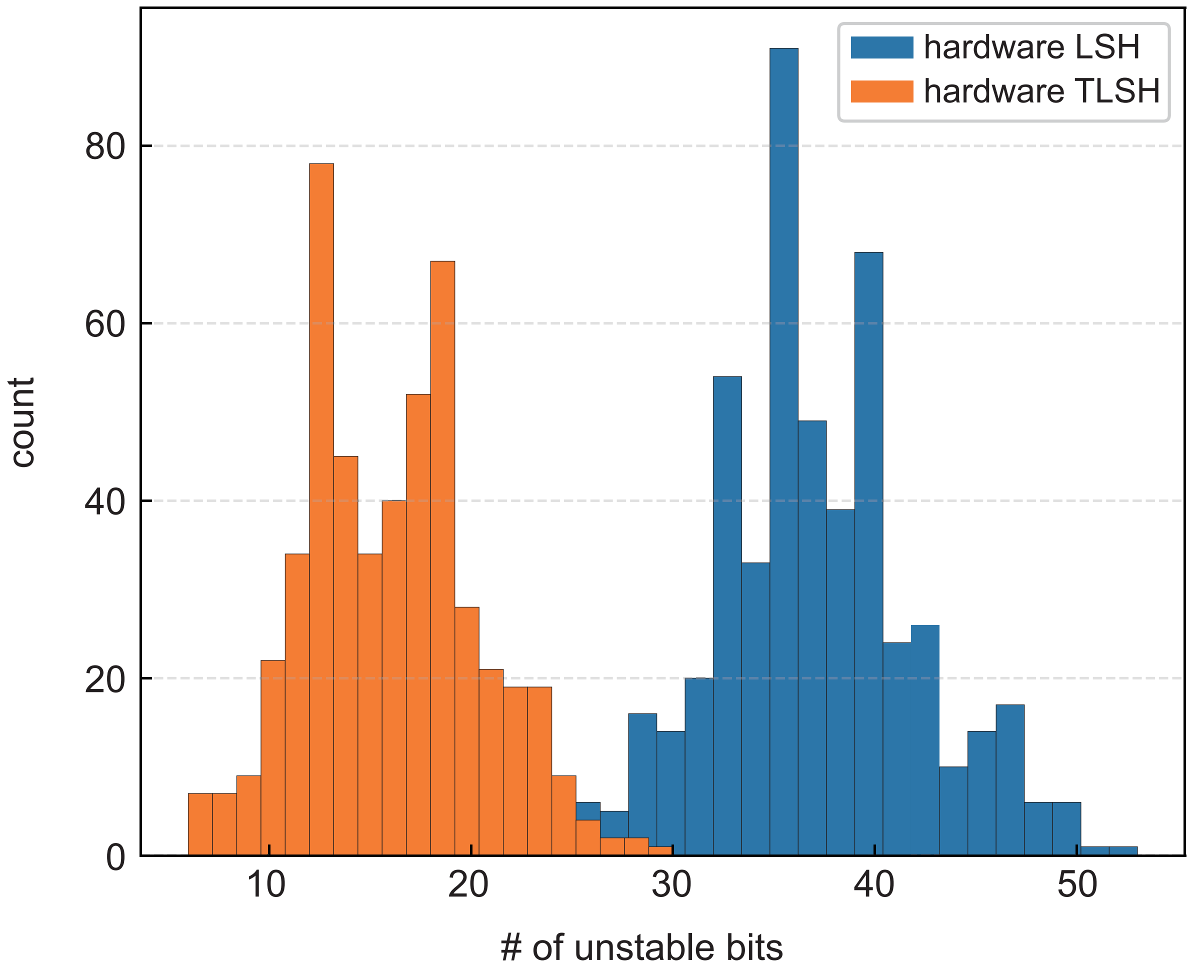

Supplementary Figure 5: Number of unstable bits using hardware TLSH and LSH. Ideally, we expect no unstable bits when performing hardware LSH so that it's equal to software LSH. However, due to many device nonidealities, there will be many unstable bits mainly caused by the conductance fluctuation. In this work, we apply TLSH to solve this problem. Here, we use the same set of vectors in the main text and repeat the hashing process 100 times with the same conductance map of size 64×129. We then count the number of unstable bits 100 times for both TLSH and LSH. The result shows that by applying TLSH we can reduce the number of unstable bits, thus increasing the robustness of our hardware in terms of hashing process.

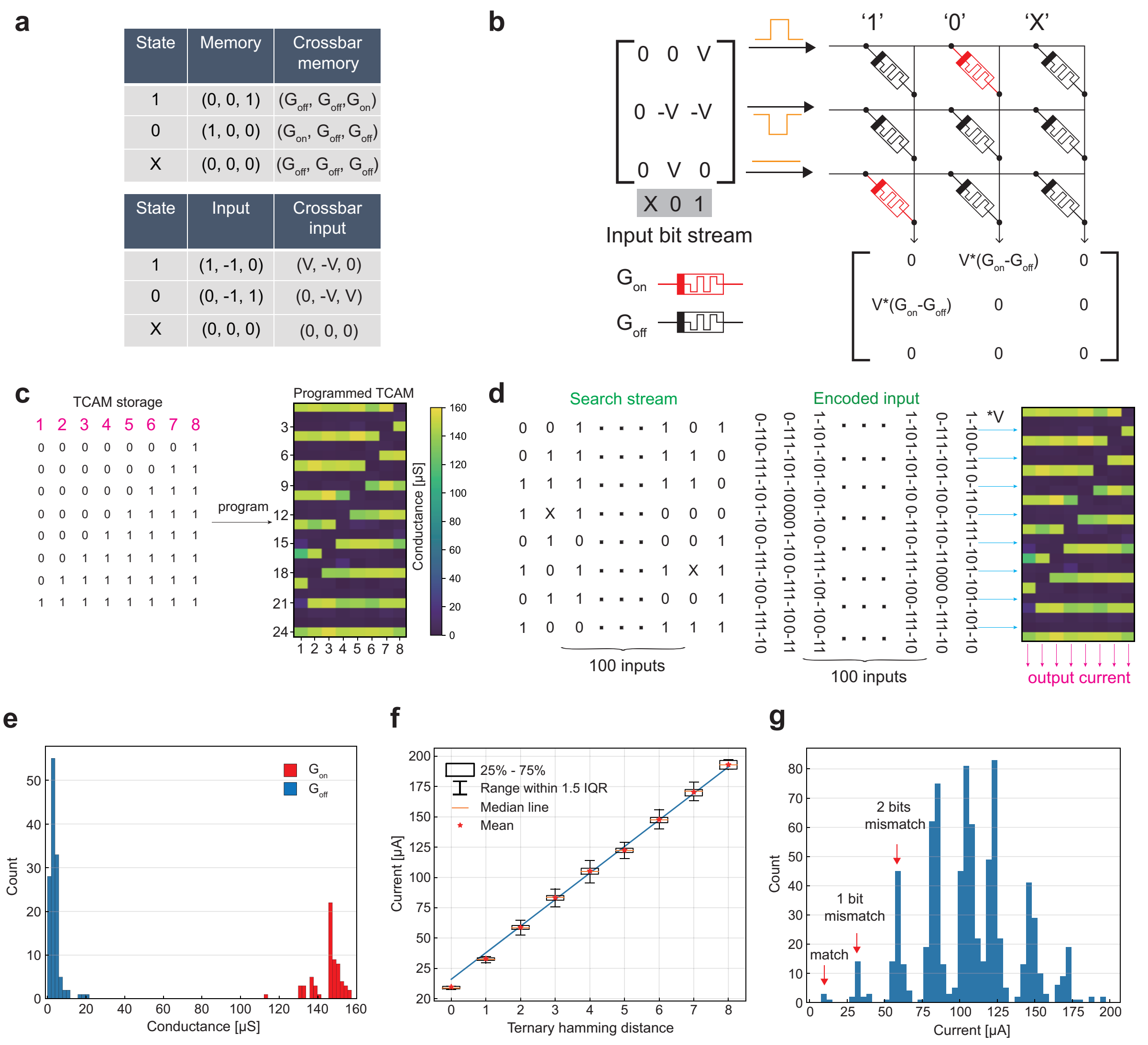

Supplementary Figure 6: 3-bit encoding method for TCAM using crossbar array. **a**, TCAM storage and input encoding method of 3-bit encoding. **b**, The schematic of experimental set-up for THD calculation by using the dot product. Three bits are stored in crossbar array using 3-bit encoding. Search bits are successively applied to the row line and the output current from the column line exhibits the THD between input and memory. **c**, Eight binary vectors stored in crossbar as memory after 3-bit encoding. **d**, 100 different ternary vectors sent to TCAM to perform THD calculation in a parallel way. **e**, Distribution of G_{on} and G_{off}. **f**, The ouput current exhibits a linear dependence on the THD. IQR, interquartile range. **g**, Current distributions are separated from each other through which we can obtain the number of mismatch bits, *i.e.* , THD.

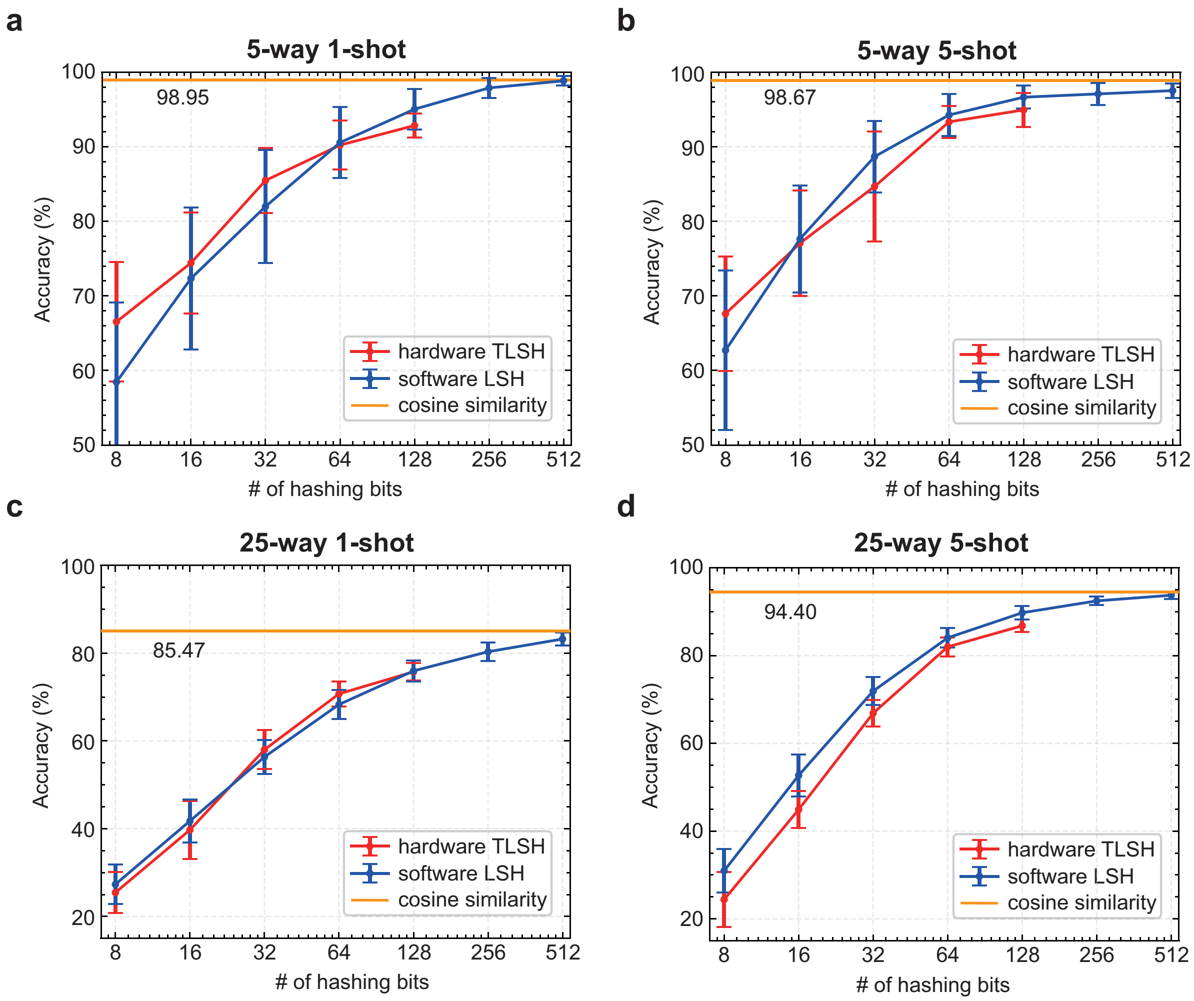

Supplementary Figure 7: Robustness of hardware TLSH on few-shot learning. a, b, c, d, To demonstrate the robustness of hardware TLSH which exploits the intrinsic stochasticity of memristors, we randomly choose 20 different conductance maps to perform the hashing process. The figure shows the classification accuracy with hardware TLSH and software LSH based on the experimentally generated hashing codes. The result exhibits the robustness of our memristor crossbar array served as hashing vector generator whose behavior is nearly the same as software LSH. Furthermore, we can obtain the same accuracy as cosine similarity on few-shot learning tasks if we scale the hashing bits up to 512 which is feasible in the prevailing crossbar architectures.

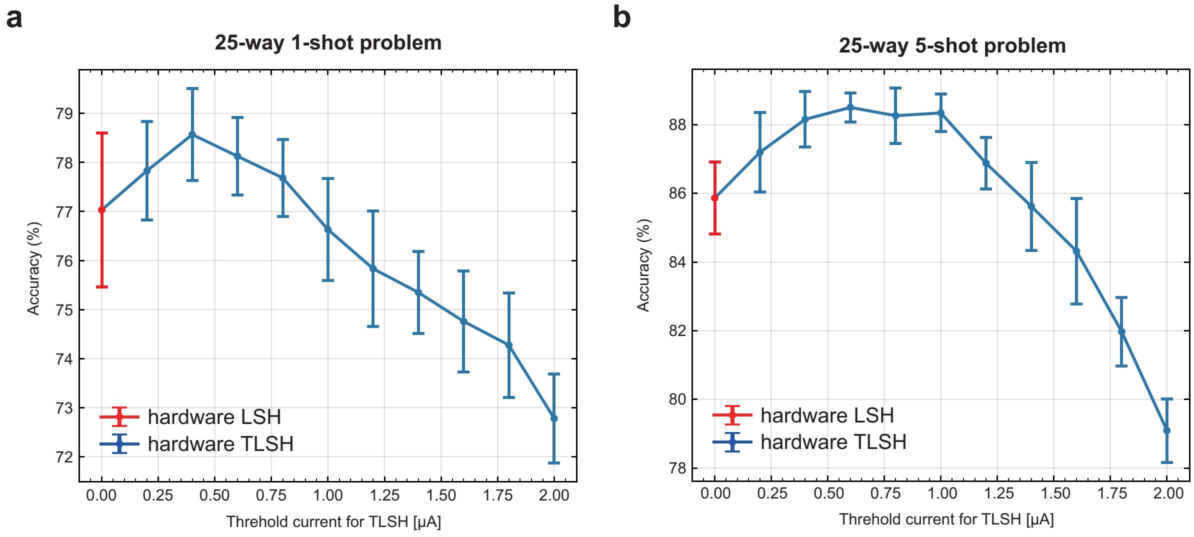

Supplementary Figure 8: Impact of the threshold current for hardware TLSH on the classification accuracy. a, b, As expected, the influence of the threshold current for hardware TLSH should be positive first and negative afterward. Because introducing the wildcard 'X' in LSH can mitigate the defect of device nonidealities but can also cast away the information of original input vectors. Here, we experimentally demonstrate the classification accuracy of 25-way problems with different threshold currents. The result shows that for our system, the performance reaches a peak with a threshold current around 0.4 μA. The zero thresholds current case denotes hardware LSH. Each task is repeated 100 times.

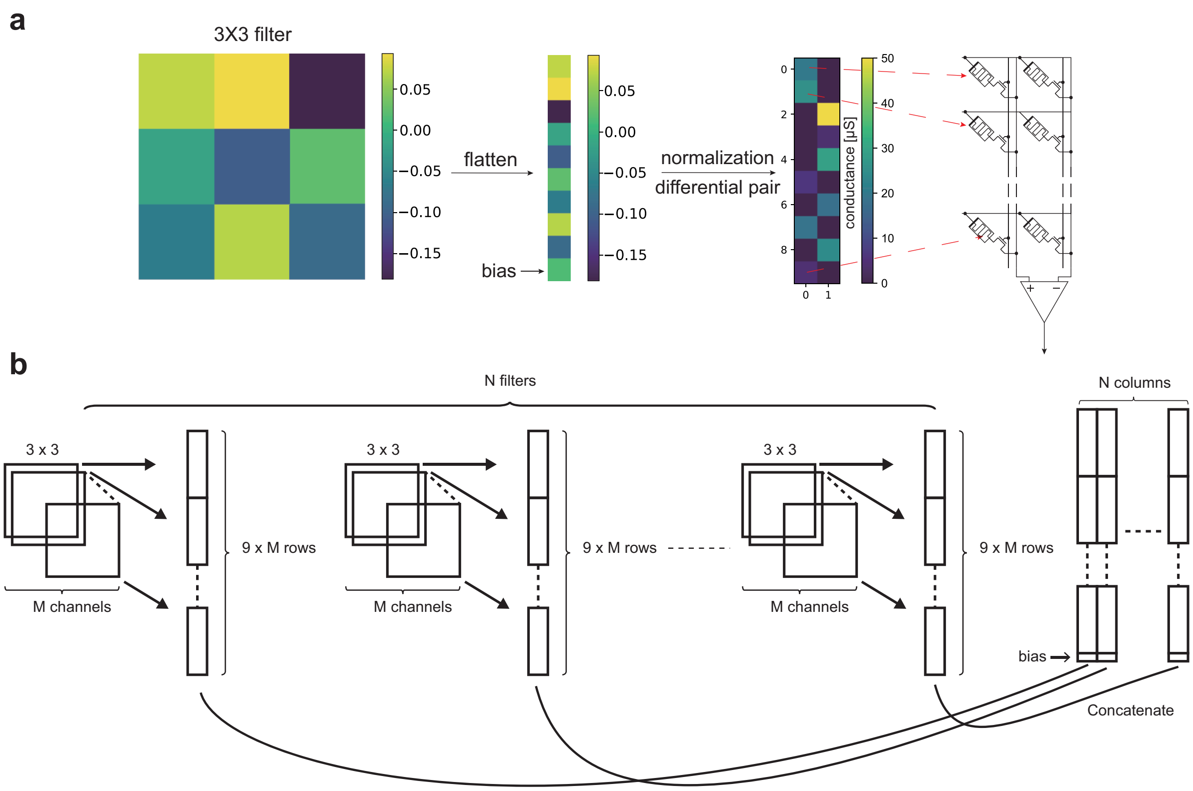

Supplementary Figure 9: Convolutional layers mapped to crossbar arrays a, A 3×3 convolutional kernel is flattened first and mapped to conductance using differential pair. **b**, N filters with each containing M channels are concatenated together and mapped to crossbar arrays. Different filters perform the convolution in a parallel way.

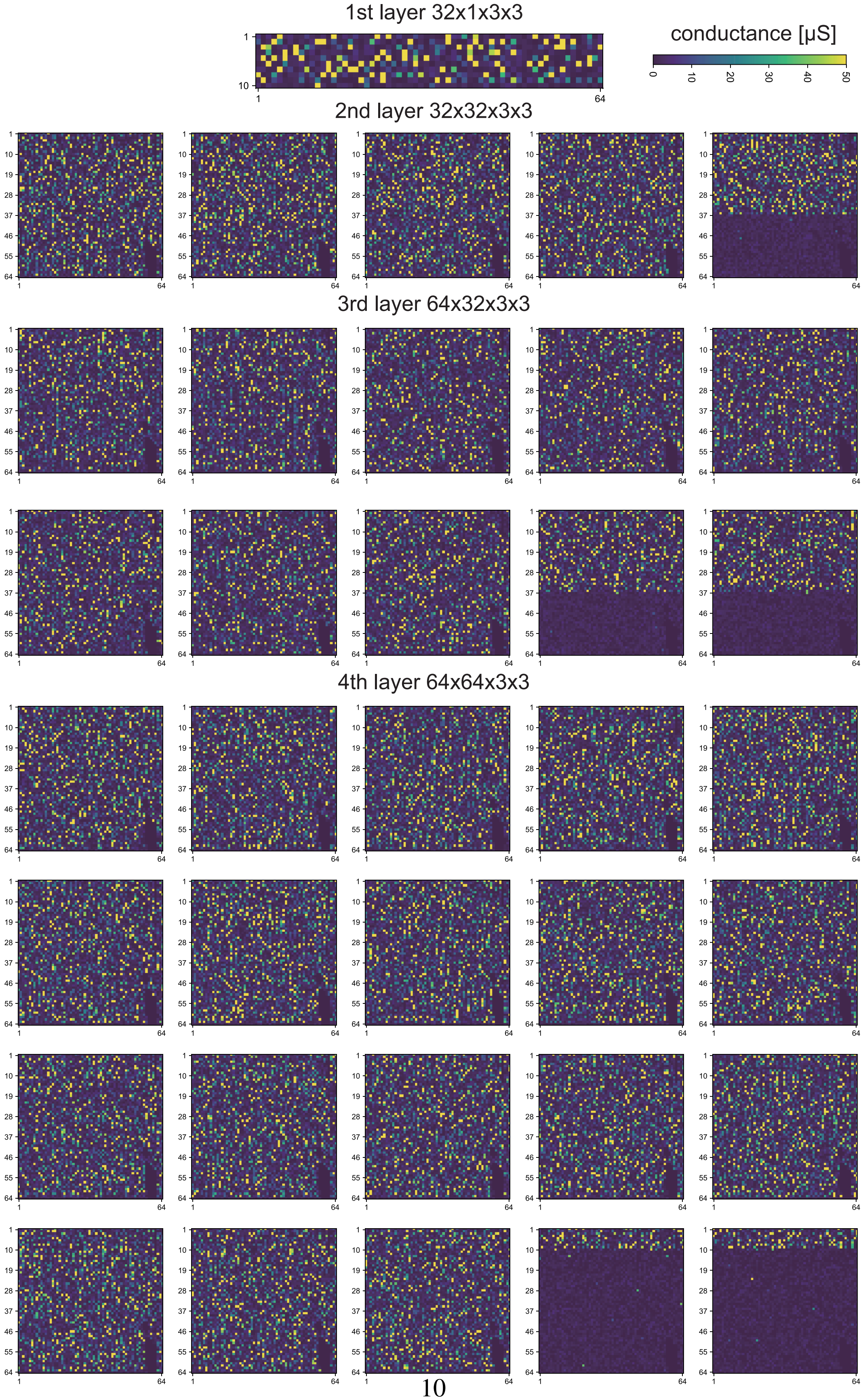

Supplementary Figure 10: Experimental conductance map of convolutional layers

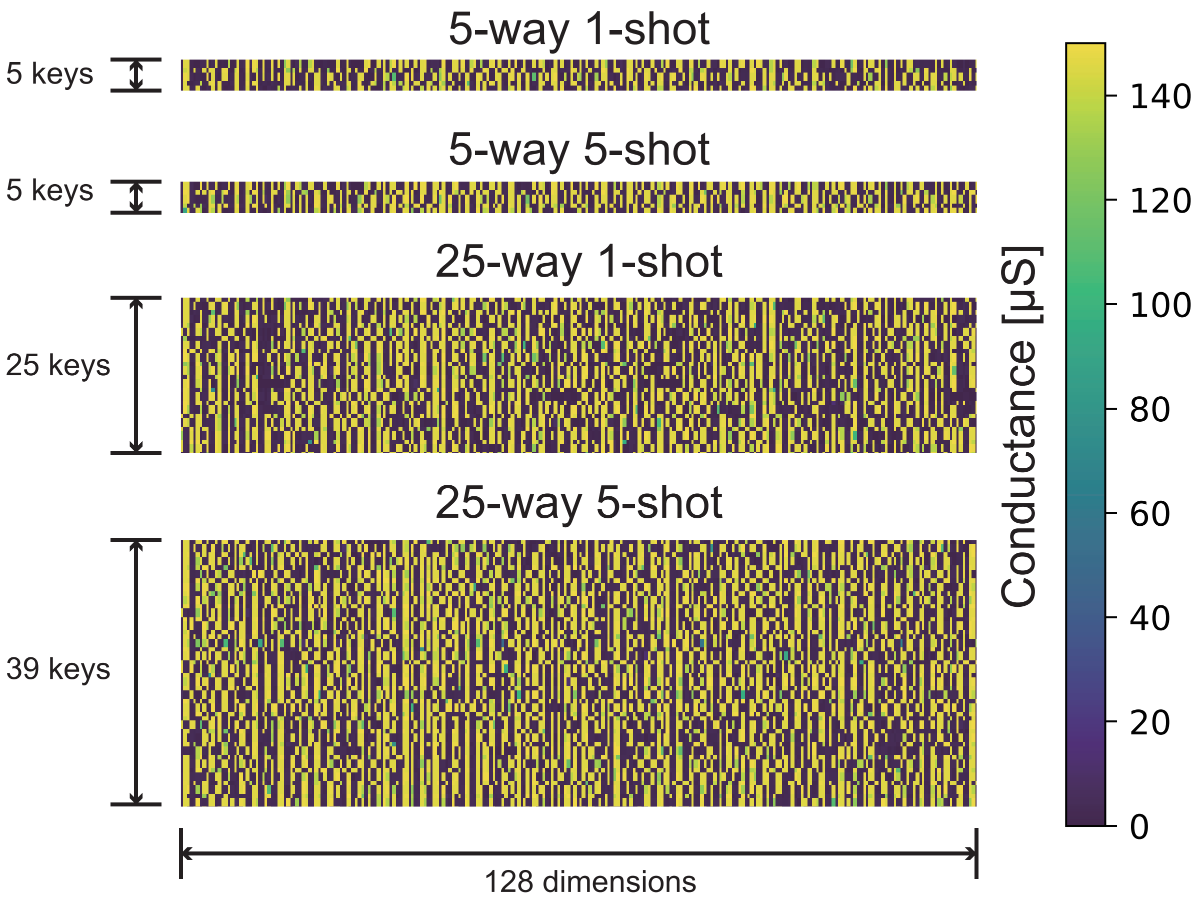

Supplementary Figure 11: Memory stored in the crossbar-based TCAM after TLSH operation and binary update process

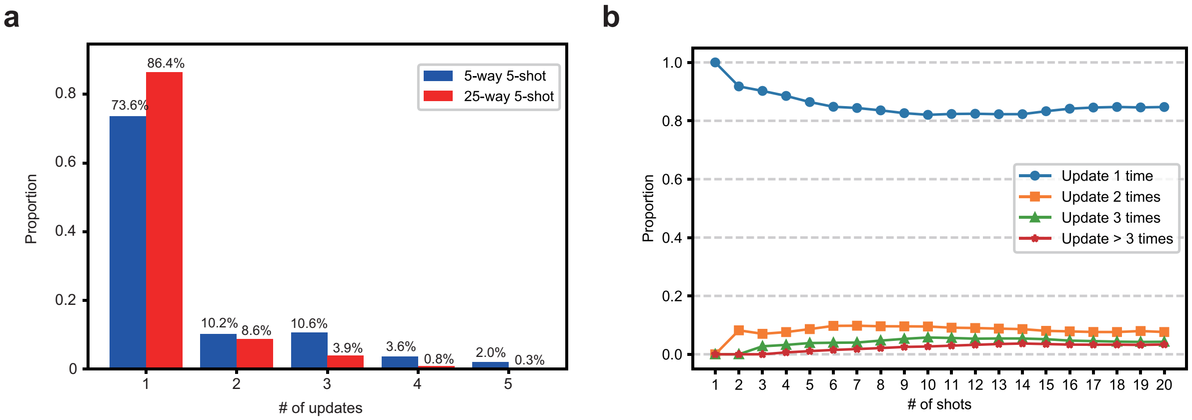

Supplementary Figure 12: Update times of the memory stored in crossbar-based TCAM of multi-shot learning. The majority of the bits are updated only once which is when writing the newly input vector into the new location of the memory. Unlike the conventional memory update method which needs to update every value in the real-valued vectors, our proposed binary update method only updates very few bits in the memory which is suitable for lifelong learning given the endurance of memristors. **a,** Statistical view of the update times of bits during standard 5-shot learning. **b,** To analyze the life-long learning properties, we perform the multi-shot learning on the 25-way task from 1-shot to 20-shot. It shows that nearly 80% of the bits stored don't need to be updated once written to store the new input vector. There are only smaller than 5% of bits that need to be updated higher than 3 times throughout the 20-shot learning.

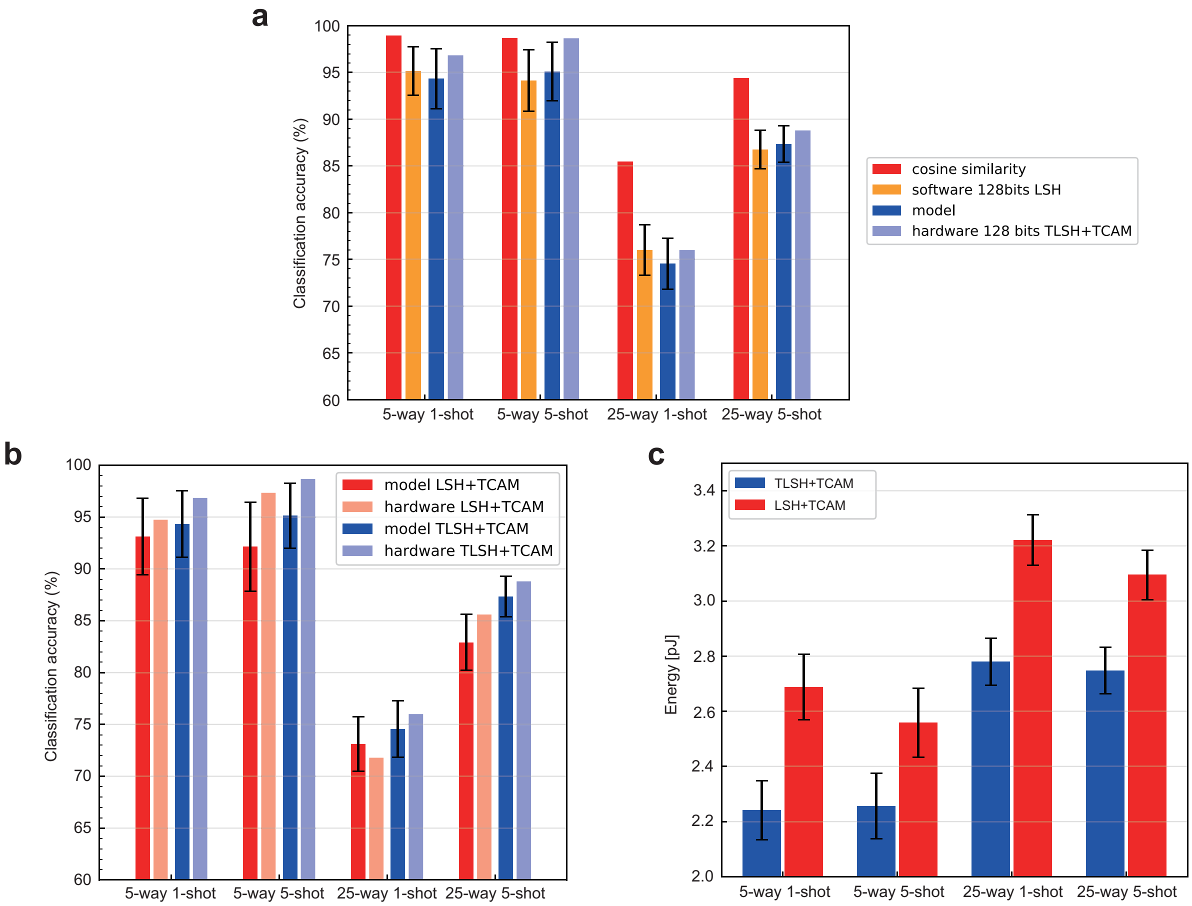

Supplementary Figure 13: Simulation results on Omniglot dataset accord with experimental results **a**, The classification accuracy of the derived memristor device model, along with cosine similarity, software-based LSH with 128 bits, and end-to-end experimental results on crossbar arrays. **b**, Comparison over LSH and TLSH with both simulation and experiment on few-shot learning tasks. **c**, Average energy consumption of each TCAM search operation with TLSH and LSH, respectively.

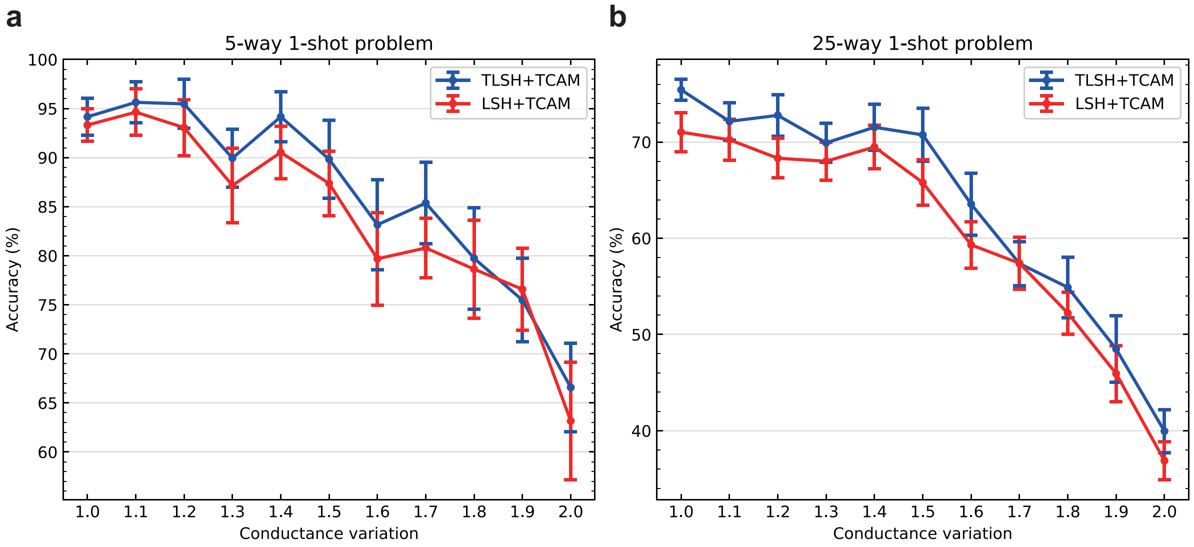

Supplementary Figure 14: Impact of device variation on classification accuracy. a, b The experimental device-to-device variation is shown in Supplementary Fig. 4**c**. In the simulation, we increase the variation to explore its influence of it on classification accuracy. The results show that for both 5-way 1-shot and 25-way 1-shot problems, the classification accuracy drops by 10% when the conductance variation is about 50% larger than the experimental data. The value of conductance variation is the value of parameter *s* in Supplementary Table 2

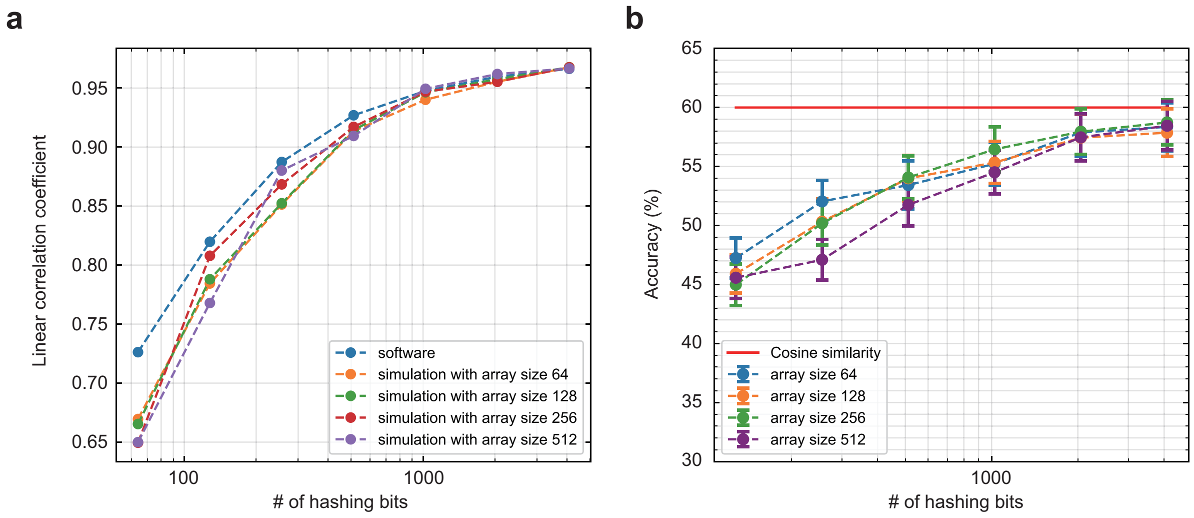

Supplementary Figure 15: Performance of scaled-up MANNs with different array size a, TLSH performance with different array size. The sneak path problem has no influence on the TLSH part because most of the devices are at the OFF state and the hyperplanes are random. **b**, Classification accuracy with different array size. The performance degradation is found with a bigger array size and a smaller number of hashing bits. This is because the sneak path problem affects the performance of TCAM, resulting in miscalculating Hamming distances. However, the accuracy remains nearly the same for different partitioned array sizes when the number of bits is large.

2 Supplementary Tables

Supplementary Table 1: Iterative write-and-verify method

Program parameter	V_1/V	V_2/V	ΔV/V
SET voltage	1.0	2.5	0.1
RESET voltage	0.5	3.5	0.05
SET gate voltage	1.0	2.0	0.1
RESET gate voltage	5.0	5.5	0.1

Supplementary Table 2: Derived values of memristor model parameters

Parameter	Description	Value
G_0	Initial conductance after program	-
a	Linear fitting parameter	0.782
b	Linear fitting parameter	-2.168
s	Device-to-device variation	0.983

3 Supplementary Notes

Supplementary Note 1. Sensing margin in crossbar-based TCAM

The sensing margin between the match and mismatch cases in conventional TCAM measures the reliability of the hardware under extreme conditions. For TCAM circuits based on emerging memory devices, a higher sensing margin also provides better tolerance to device variation and thus a smaller bit error rate.

In this work, our crossbar-based TCAM does not only distinguish between match and mismatch cases but also returns the number of mismatches. For few-shot learning with MANN, what is required is distinguishing between the closest match and the next close match. Here we describe the sensing margin β for our crossbar-based TCAM in detecting the closest match in Equation 1.

$$\beta = \frac{I_{m+1} - I_m}{I_m} = \frac{I_{m+1}}{I_m} - 1 \tag{1}$$

where I_m is the largest possible current (or voltage after the sensing amplifier) of the closest match, and I_{m+1} is the smallest possible current (or voltage after the sensing amplifier) of the next closest match. A higher sensing margin β indicates fewer errors in detecting the closest match, which is a function of a variety of parameters, including the memristor device ON/OFF conductance ratio $r = G_{\mathrm{ON}}/G_{\mathrm{OFF}}$, word length (the width of the array) N, the number of mismatches of the closest match M, the number of wildcard 'X' in query vector/memory entry K, etc. The following analyses aim to reveal the trade-off relationship among the parameters. The effect of device variation and wire resistance is not taken into consideration for simplicity.

- **Case 1**

  We start with the simplest case, where there is no wild card 'X' in the query vector, and we want to detect the exact match between the query and keys with a word length N. The sense margin can be written as:

  $$\beta = \frac{I_{m+1}}{I_m} - 1 = \frac{G_{on}V_s + (N-1)G_{off}V_s}{NG_{off}V_s} - 1 = \frac{r-1}{N} \tag{2}$$

  where V_s is the search voltage, and other parameters are defined previously.

  One finds that the sensing margin increases with the memristor ON/OFF ratio r and decreases with the word length N. On the other hand, if we want the sense margin to be at least β_0, the word length needs to be smaller than $\frac{r-1}{\beta_0}$. For example, if the ON/OFF ratio (r) is 100, and the sense margin should be at least 0.5, the maximum word length $N_{\max}$ is limited to $\frac{100-1}{0.5} = 198$.

- **Case 2**

  Instead of detecting the exact match, here in this case study, we detect the closest match (with M mismatch bits). Wildcard 'X' is also ignored in this case. The sense margin can be written as:

$$\beta = \frac{I_{m+1}}{I_m} - 1 = \frac{(M+1)G_{on}V_s + (N-M-1)G_{off}V_s}{MG_{on}V_s + (N-M)G_{off}V_s} - 1 \tag{3}$$

$$= \frac{(M+1)r + N - M - 1}{Mr + N - M} - 1 \tag{4}$$

$$= 1/\left(M + \frac{N}{r-1}\right) \tag{5}$$

  In addition to the trade-offs revealed in Case 1, one also finds that the sensing margin β also decreases with the number of mismatch bits M. So, a fewer number of mismatch bits is preferred for a given application.

- **Case 3**

  Here, we consider the case that the query hash code has K wildcard bit 'X' from the TLSH step, and we want to detect the closest distance with M mismatch bits between the query and keys with a word length of N. The sense margin can be written as:

$$\beta = \frac{I_{m+1}}{I_m} - 1 = \frac{(M+1)G_{on}V_s + (N-M-K-1)G_{off}}{MG_{on}V_s + (N-M-K)G_{off}V_s} - 1 \tag{6}$$

$$= \frac{(M+1)r + N - M - K - 1}{Mr + N - M - K} - 1 \tag{7}$$

$$= 1/\left(M + \frac{N-K}{r-1}\right) \tag{8}$$

  It is expected because introducing the wild card equivalently reduces the word length (ignoring the wire resistance effect). From Equation 8, one concludes that the sense margin for the closest match increases with decreasing mismatch bits M and wordlength N, and rising number of wildcard K and device ON/OFF ratio r. In addition, when we introduce 'X' in the hashcodes of the query, the number of mismatch bits M of the nearest neighbor is usually reduced, further increasing the sensing margin.

From the above case study, we conclude that both decreasing the number of mismatch bits M and increasing the number of the wild card bits K improve the sense margin and robustness of the search operation. The ternary locality sensitive hashing (TLSH) that we proposed in this work

achieves both goals at the same time while maintaining the same accuracy. The main reason behind this is that hashcodes generated by random projections have some redundancy bits that can be cast away by assigning them to the wildcard bit 'X'. Here we show the statistical results extracted from the few-shot classification to demonstrate the idea.

We first analyze the statistics of the mismatch bits numbers (or hamming distance) of the closest match M during the few-shot learning experiments. The distribution of the hamming distance is shown in Supplementary Fig. 16**a**. The result shows that by introducing 'X' in the query hashcodes, the number of mismatch bits M of the closest match decreases significantly. The experimental sensing margin is then extracted from the output current of the closest match (the smallest current I_0) and the next close match (the 2nd smallest current I_1), by the equation: $\beta = \frac{I_1}{I_0} - 1$. As expected, the experimental sensing margin is inversely proportional to the the hamming distance, *i.e.* $\beta \propto \frac{1}{M}$ as is shown in Supplementary Fig. 16**b**. Though the sense margin here is not the worst sense margin characterized by Equation 8, we still see a similar trend. As a result, the sensing margin is improved by introducing the wildcard 'X' from the TLSH because of the reduced hamming distance of the closest match. The conclusion is clearly demonstrated in the experimental sensing margin distribution shown in Supplementary Fig. 16**c**. Specifically, the probability that the sense margin is larger than two with TLSH is more than two times higher than that using LSH. In light of the above analysis, we can conclude that the reduced mismatch bit number from our TLSH scheme improves the sense margin of the crossbar-based TCAM for the closest match search.

Similar phenomena can also be observed in a scaled problem for few-shot learning with Mini-ImageNet dataset. The result in Supplementary Fig. 17**a** shows that the sensing margin decreases with the word length N, and the sensing margin with TLSH is always higher than that with LSH, because of the introduced wildcard bit 'X'. Supplementary Fig. 17**b** also show that the introduction of the wildcard 'X' also reduces the number of mismatch bits, further improving the sensing margin.

Note that our TLSH method can also combine with other TCAM structures like 2T-2R[1,2], 2Flash[3] 2FeFET[4,5] which have the similar characteristic, but use latched voltage signal as the output. As mentioned, the sense margin of our crossbar-based TCAM can be further improved with the 3-bits encoding method, as discussed in Supplementary Fig. 6.

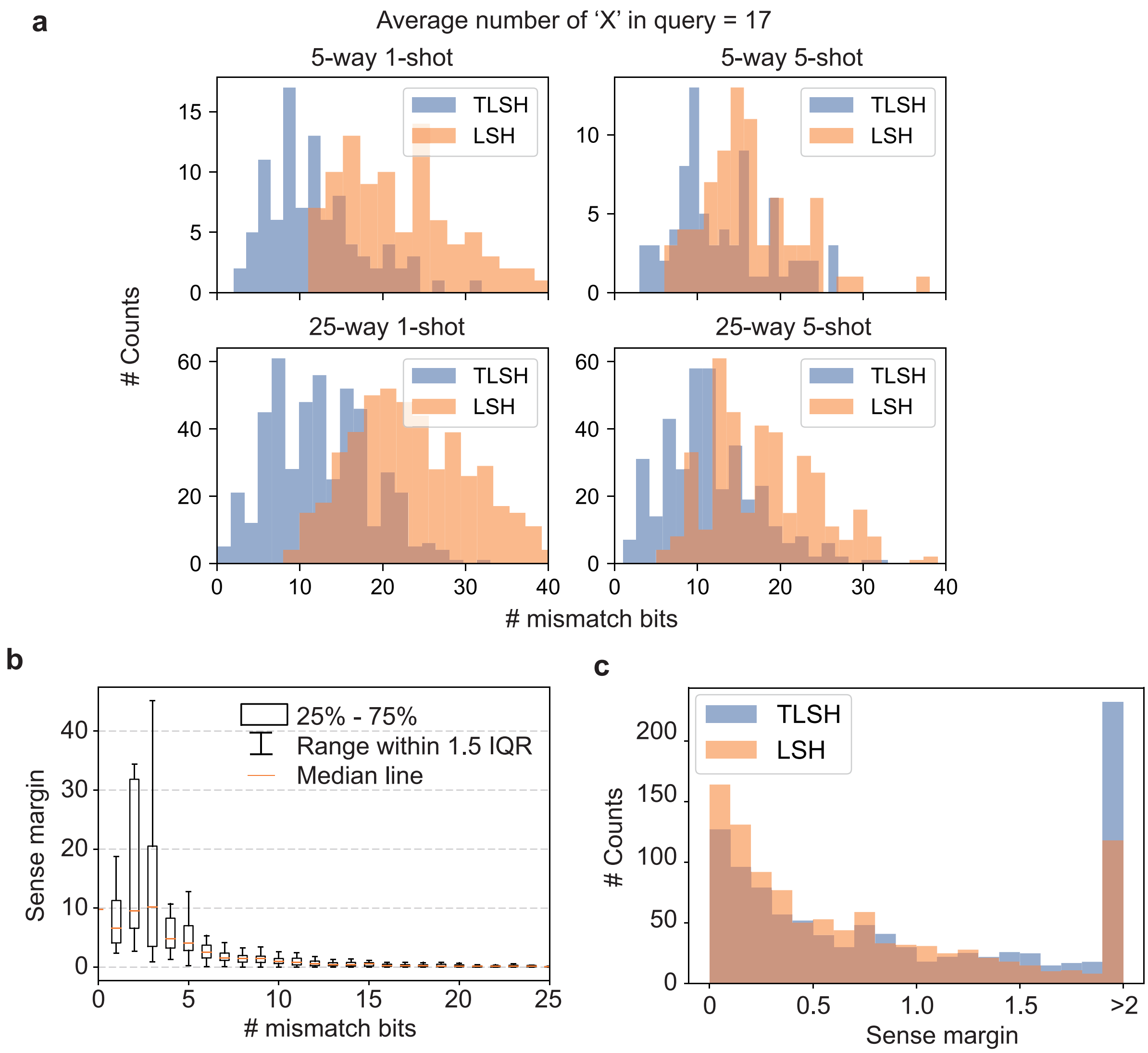

Supplementary Figure 16: Sense margin analysis of the few-shot classification on the Omniglot dataset. a, Distribution of the mismatch bits of the closest match between query and keys in 4 different tasks. We can see that by using TLSH in the hashing operation, we can significantly decrease the number of mismatch bits. **b,** Relationship between the sense margin and the number of mismatch bits M of the closest match measured in 4 tasks. The sense margin decreases as the M increases. **c,** Distribution of the sense margin for TLSH and LSH. The probability that the sense margin is larger than 2 using the TLSH method is twice as high as that using LSH. This verifies that our TLSH method can significantly increase the sense margin.

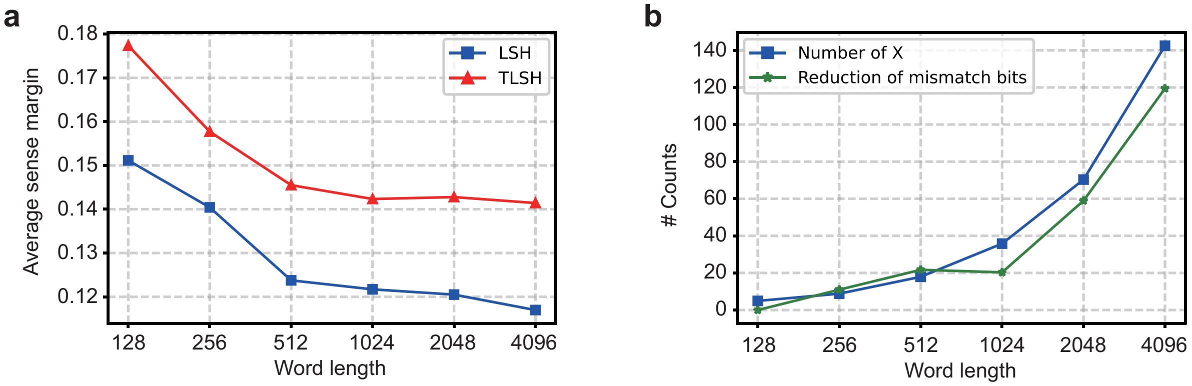

Supplementary Figure 17: Sense margin analysis of the 5-way 1-shot classification on the Mini-ImageNet dataset. a, Average sense margin drops as the word length increases. Sense margin on the hashcodes generated by TLSH method is always higher than that by LSH method. **b,** Reduction of mismatch bits grows with the word length and follows a similar trend as the number of 'X' in the query.

Supplementary Note 2. Peripheral circuit design for ternary locality sensitive hashing

To demonstrate that the ternary sensing scheme for the ternary locality sensitive hashing can be achieved in hardware with minimum overhead, we design the peripheral circuits for the TLSH shown in Supplementary Fig. 18**a**. Our design is far from optimal yet, but it demonstrates that crossbar-based TLSH with a simple design can also achieve the required function with compelling latency and energy benefits.

In this design, the output currents of every two adjacent columns are firstly transferred into voltage signals using trans-impedance amplifiers (TIAs) and then the voltage difference is calculated using a subtractor. After that, the TLSH sensing block is used to generate the ternary output based on the differential signal, which is composed of three comparators, three multiplexers, and one inverter. To minimize the energy consumption of the TLSH sensing block which is the major energy bottleneck compared with the LSH sensing method, we choose to use the StrongArm latch comparator[6] which features zero static power and can make decisions within minimum latency ($< 1\,\text{ns}$).

As illustrated in the Supplementary Fig. 18**a**, the *Comp1* first compares the differential signal with the reference voltage to give the sign of the hashing output when the *clk1* goes high. *Comp2* and *Comp3* serve as the "ternary" part and compares the signal with the threshold V_{th} once *clk2* goes high. The multiplexer is controlled by the output of the *Comp1* to choose which signal to pass from *Comp2* and *Comp3*. If the differential signal is within the threshold, as the TLSH algorithm required, the sensing circuit should output an 'X'. In the circuit, the *Vx* goes high in this case and thus puts the differential logic signal from two multiplexers in (0, 0). If the differential signal exceeds the threshold, the output is only dependent on the output of *Comp1* which is the sign of the signal, representing the original binary hashing code. Supplementary Fig. 18**b, c** shows the detailed design schematics of the operational transconductance amplifier (OTA) for TIAs, subtractors and the StrongArm latch, respectively.

To prove the functionality of the TLSH sensing circuit, we simulated the design in Cadence Virtuoso following 180 nm technology design rules from a commercial foundry. The simulated transient response of the TLSH peripheral circuit is shown in Supplementary Fig. 18**d**. The input consists of three serial voltage pulses that represent the output from the crossbar, corresponding to '1', 'X', and '0', respectively. From the differential output (V_{o1} and V_{o2}), we can see that the 2-bit logic signal can well represent the input $(1 \rightarrow (1,0), X \rightarrow (0,0), 0 \rightarrow (0,1))$. This demonstrates the circuit can make the right decision based on the differential signal and the threshold voltage within 1 ns. We further measured the power of the TLSH block within 1 cycle and estimated about 0.44 mW.

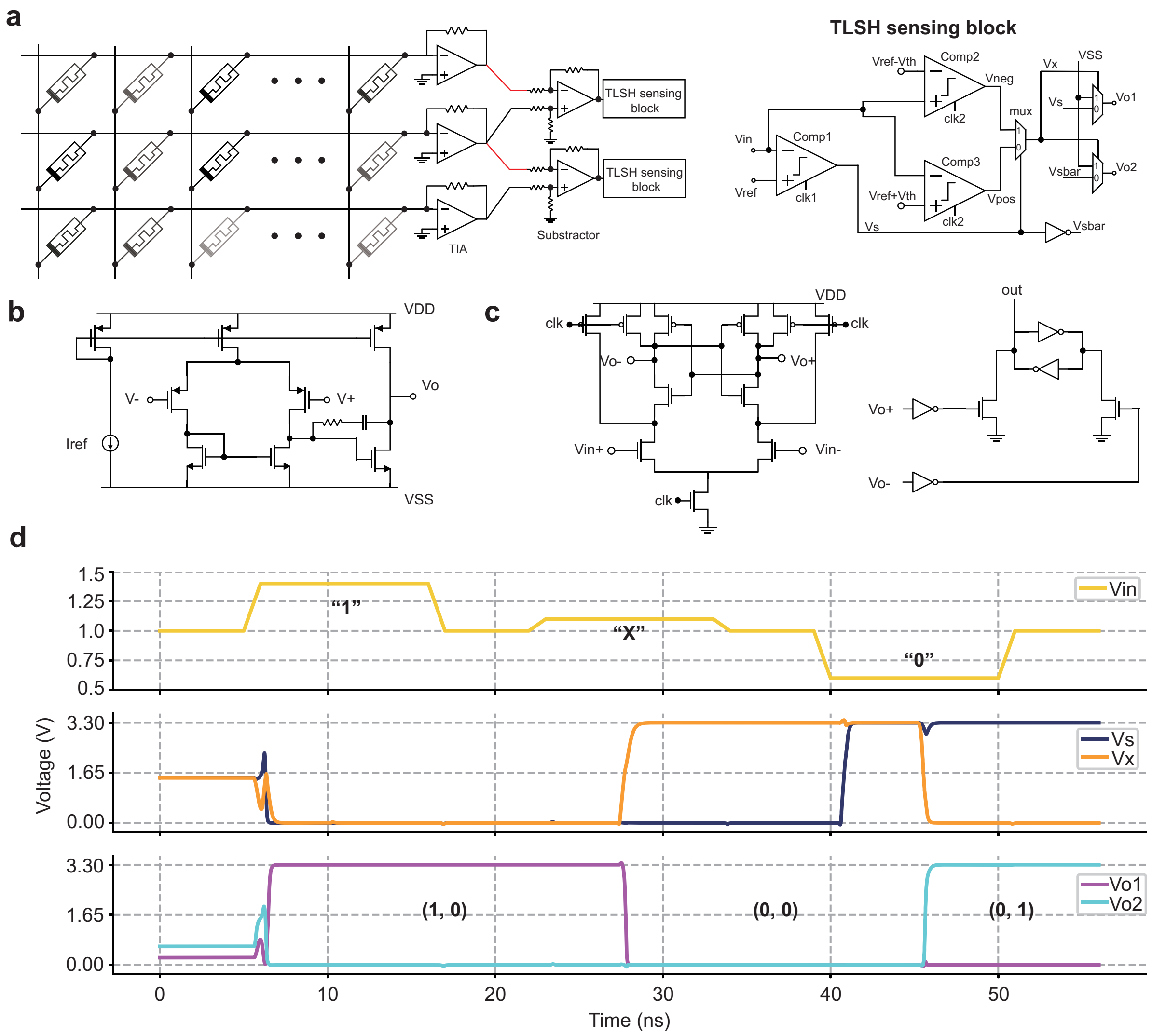

Supplementary Figure 18: The peripheral circuit design and simulation results for crossbar-based TLSH a, Schematic of the peripheral circuits for sensing ternary hashing codes. Every two adjacent columns are attached to two transimpedence amplifiers (TIAs), one subtractor and one TLSH sensing block. Both the TIA and subtractor are based on an operational transconductance amplifier (OTA). The TLSH sensing block consists of three StrongArm latch dynamic comparators, three multiplexers (Mux), and one inverter. **b,** The detailed design of OTA in TIAs and subtractors. **c,** Schematic of the StrongArm latch comparator, which consists of a comparator and a reset-set latch (RS-latch). **d,** Simulated transient response of the TLSH sensing block. The input voltage contains three states ("1", "0", and "X") that need to be detected. The differential outputs from two multiplexers show that it can distinguish three states with a 2-bit representation ("1, 0", "0, 0", and "0, 1"). The location of the signals is labeled in **a**.

Supplementary Note 3. Peripheral circuit design for crossbar-based TCAM

We have demonstrated that the TCAM implemented by the crossbar can compute the Hamming distances between the input query and stored keys in a single time step, but the current can be significant when the hamming distance is high and with a large crossbar array, leading to large power consumption and analog computing error. On the other hand, the memory augmented neural network does not require the associative memory to output the full range of mismatched numbers, and in most cases, only the 'match' or 'closest match' needs to be found. Therefore, it is desired to suppress the current for unnecessary sensing of a large hamming distance for lower energy consumption.

To achieve this goal, we propose two different circuit designs, one is the threshold sensing and the other is the voltage mode sensing, as detailed below.

a) Threshold sensing

The schematic of the threshold sensing is shown in Supplementary Fig. 19**a**. The idea is to use a Schmitt trigger to detect whether the output from the TIA exceeds a threshold voltage. Once the output reaches the threshold *Vth*, the output from the Schmitt trigger turns to a high logic and thus turns off all the transistors on that row. It floats that row and shuts off the current. After that, the output from the TIA goes back to *Vb* (a virtual ground) again. But since there is hysteresis in the Schmitt trigger, *Vcp* changes to a lower voltage to keep the transistor off. When there is another new input coming, *Vref* will first go to VDD to let the transistor on and then goes back to reference voltage for another calculation. The threshold voltage of the Schmitt trigger can be calculated as:

$$Vth = Vref\frac{R1}{R1+R2} + VDD\frac{R2}{R1+R2} \tag{9}$$

After shutting down the transistor, *Vcp* becomes $Vref\frac{R1}{R1+R2}$ which should be designed to be lower than *Vb*. When a new input pulse coming, the *Vref* needs to be set to VDD first to push the *Vcp* to $VDD\frac{R2}{R1+R2}$ which should be designed to be higher than *Vb*.

We implemented and simulated the circuit in the Cadence Virtuoso under a 180 nm technology node from a commercial foundry as shown in Supplementary Fig. 19**b**. From the figure we can see that once the *Vo* is higher than a predefined *Vcp*, which represents the maximum hamming distance we want to detect, the current on the memristor I_{on} will be shut down to 0 A, saving the energy consumption. Supplementary Fig. 19**c** further shows the output voltage from TIAs and the current on memristors with different hamming distances. From Supplementary Fig. 19**d**, one finds that the duration of the current is smaller with an increasing number of mismatch bits. When the signal is smaller than the threshold representing the maximum hamming distance, the signal will

be latched via a sample and hold circuit and convert to a digital signal via a low-bit count ADC.

Here we analyze how much energy it can save by using the threshold sensing technique. Assume the bandwidth of the amplifier is ω_p and the smallest pulse width t_0 is needed to achieve 1% error at the output of the TIAs, we can have:

$$\omega_p t_0 = \ln 100 \approx 4.6 \tag{10}$$

Let the N be the word length, M the number of mismatch bits, and N_{th} the threshold hamming distance, we can calculate the duration of the current when $M > N_{th}$ as follows:

$$M(1 - e^{-\omega_p t}) = N_{th} \tag{11}$$

$$\Rightarrow 1 - e^{-\omega_p t} = \frac{N_{th}}{M} \tag{12}$$

$$\Rightarrow \omega_p t = \ln(\frac{M}{M - N_{th}}) \tag{13}$$

$$\Rightarrow t = \frac{\ln(\frac{M}{M - N_{th}})}{4.6} t_0 \tag{14}$$

Note that the threshold N_{th} should be set to be a little bit higher to make sure the line with the smallest hamming distance won't be shut off.

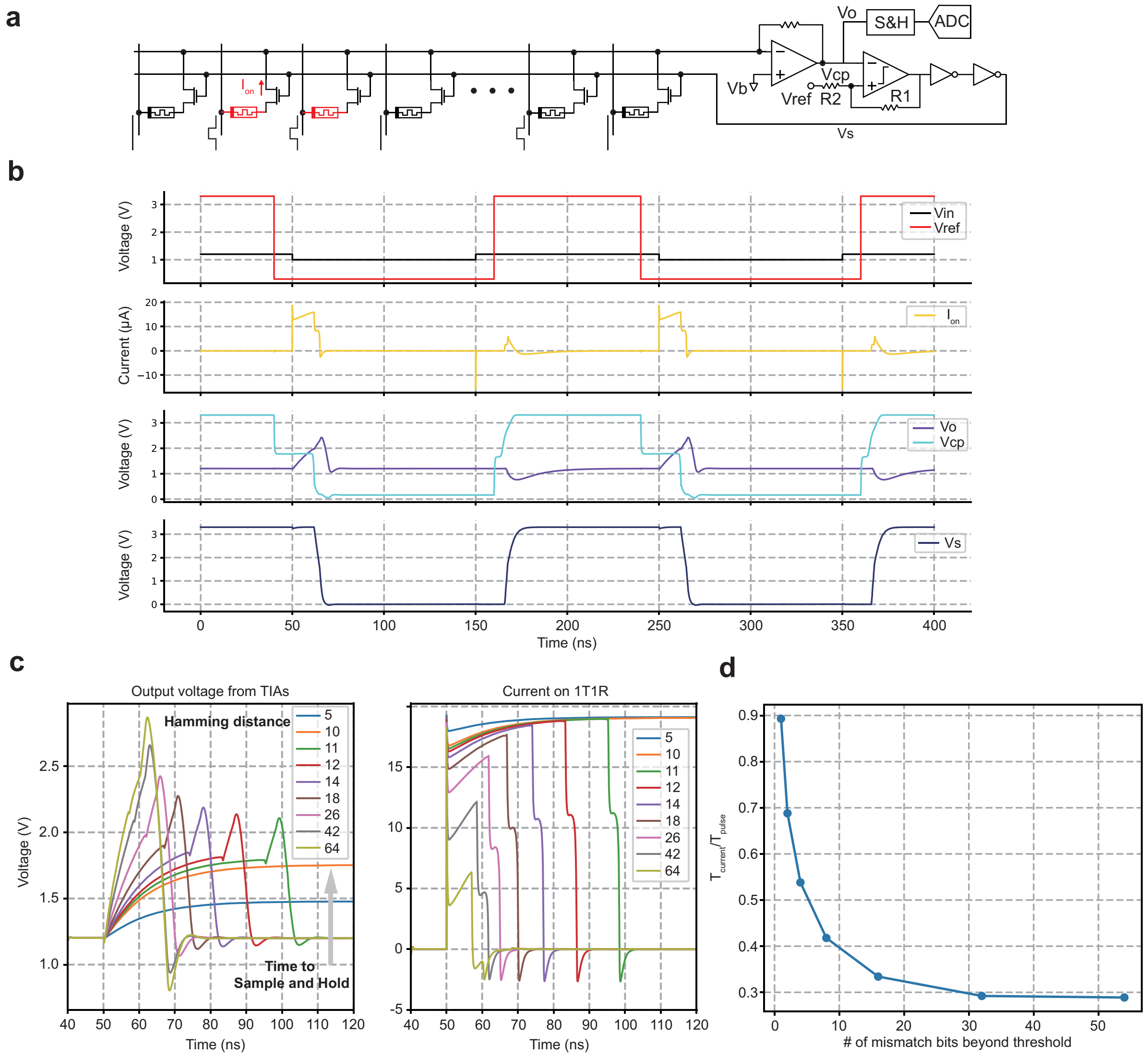

Supplementary Figure 19: The circuit design and simulation for threshold sensing technique. a, Schematic of the threshold sensing technique. A Schmitt trigger is added after the TIA to detect the current on the memristors. Two inverters are used to speed up the decision. **b,** Transient response of the threshold sensing. The labels are consistent with that in **a**. **c,** TIAs' output and current on memristors under different hamming distances. The signals corresponding to the hamming distances that are larger than the threshold value (10) are shut down before starting the sample and hold. **d,** The relationship between the ratio of the duration of current to the smallest pulse width needed ($T_{current}/T_{pulse}$) and the number of mismatch bits beyond the threshold (we set it to 10 bits in the simulation).

b) Voltage mode sensing

We explored another type of peripheral circuit design for the crossbar-TCAM to further lower the power and energy consumption. Here we use the switched capacitor amplifier to sense the output voltage from a single row. Unlike conventional current-mode sensing using TIAs, voltage mode sensing is a process of charging the capacitors. Therefore, the energy consumption is almost constant on the memristors and no longer increases with larger word length and hamming distance.

Supplementary Fig. 20**a** shows the schematic of the voltage mode sensing circuit for the crossbar-based TCAM. As shown in the figure, the sensing circuit is composed of one OTA, two capacitors C_S and C_H, and three NMOS switches. C_S is the sampling capacitor and C_H is the holding capacitor. At the sampling phase, the *clk1* and *clk2* are set to high while clk3 to low, and the output from the crossbar-TCAM is sampled on the capacitor C_S. At the holding phase, *clk3* goes high while clk1 and clk2 low, the charge on the C_S are transferred on the C_H and the output is amplified at the output of the OTA. The amplify ratio is defined as C_S/C_H.

To demonstrate the feasibility of the circuit, we simulated the circuit in the Cadence Virtuoso using a 180nm PDK from a commercial foundry. The transient response of the circuit is shown in Supplementary Fig. 20**b**. In the simulation we set the $C_S = 1\,\mathrm{pF}$ and $C_H = 100\,\mathrm{fF}$. From Supplementary Fig. 20**c** one finds that the resulting voltage on the capacitor C_S and C_H can present the calculated hamming distance from the crossbar-based TCAM.

Finally, we evaluate the energy consumption with the voltage mode sensing circuit. The voltage on the V_S during the sampling can be calculated as:

$$V_S = \frac{MV_{in}}{N}(1 - e^{-\frac{NG_{on}}{C_S}t}) \tag{15}$$

where M, N, N_{th} are the same as those in analyzing the threshold sensing technique, V_{in} is the search voltage, V_S the sampled voltage on C_S, and G_{on} the conductance in on state. If we can tolerate 1% error at the output, the time to sample the voltage is: $t_s = 4.6\frac{C_S}{NG_{on}}$. Then the energy can be calculated in the following equation:

$$E = \int_0^{t_s} (NG_{on}V_{in}^2 - V_{in}NG_{on}V_S)dt \tag{16}$$

$$= NG_{on}V_{in}^2 t_s - MV_{in}^2 G_{on}(t_s - \frac{NG_{on}}{C_S}) \tag{17}$$

$$= (4.6 - 3.6\frac{M}{N})V_{in}^2 C_S \tag{18}$$

Finally, we analyze how much energy can be saved after using these two sensing techniques. For the threshold sensing, we choose $t_0 = 10\,\mathrm{ns}$, $V_{in} = 0.2\,\mathrm{V}$ and $N_{th} = 30$ to make sure it covers the

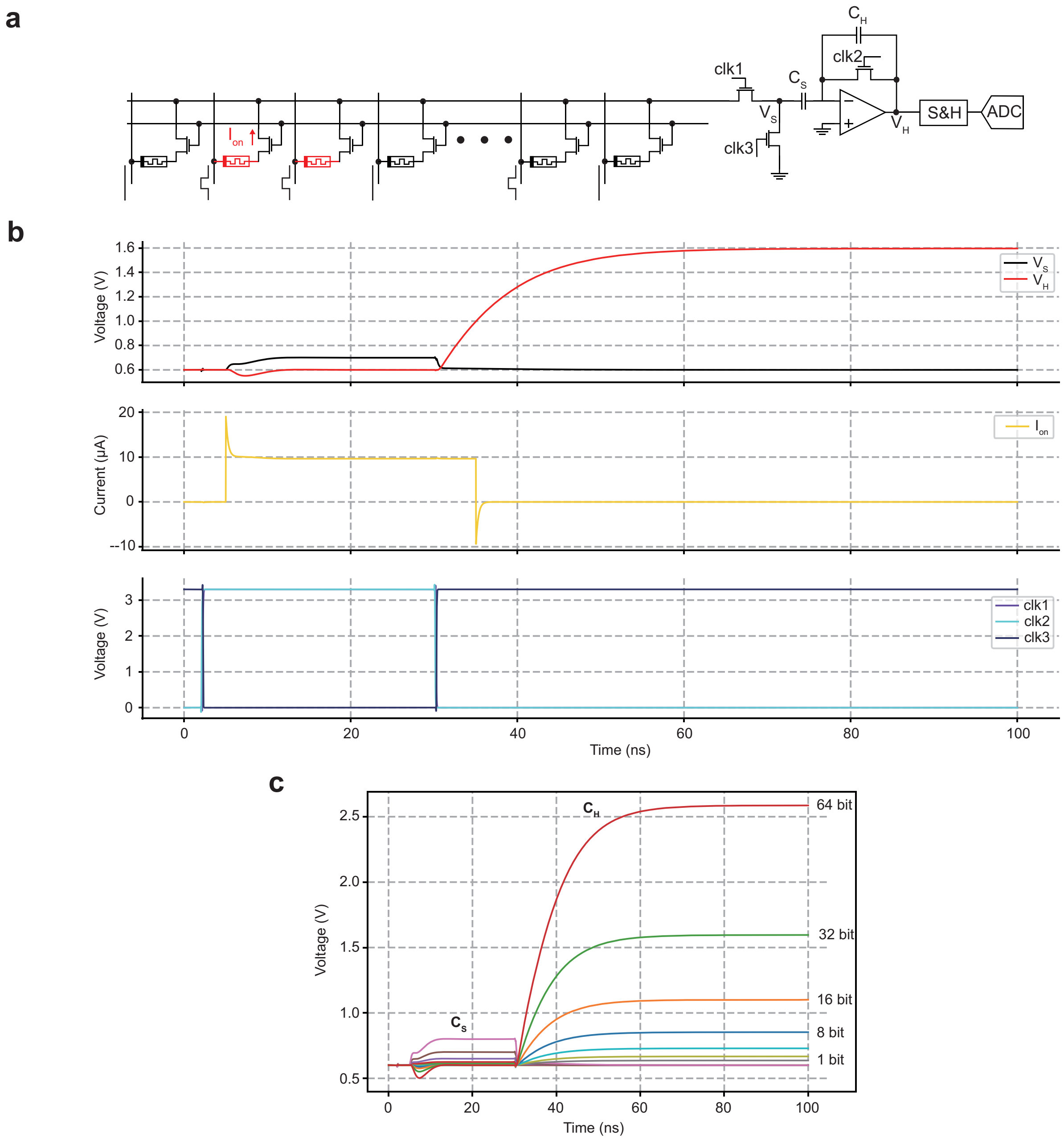

Supplementary Figure 20: The peripheral circuit design and simulation for voltage mode sensing technique. a, Schematic of the voltage mode sensing. It contains an OTA, two capacitors (one for sampling and one for holding), and three MOS switches. **b,** Transient response of a single sample-hold cycle. The voltage is first sampled on C_S and then amplified using C_H. **c,** Transient response of the voltage on C_S and C_H under different number of mismatch bits.

smallest hamming distance throughout the search operation in 5-way 1-shot task. For the voltage mode sensing, we choose $C_S = 1\,\text{pF}$ for the word length of 128. The energy consumption on the memristors with the two different types of peripheral circuits and the original are estimated and compared in Supplementary Table 3. Note that the energy consumption can be further lowered by using smaller V_{in} without degrading much accuracy.

Supplementary Table 3: TCAM search energy on memristors with sensing circuits

	Original	Threshold sensing	Voltage mode sensing
Search energy on entire memory (pJ)	11.20	4.12	0.74
Search energy per key (pJ)	2.24	0.82	0.15
Search energy per bit (fJ)	17.50	6.43	1.15

Supplementary Note 4. Energy and latency estimation

In this session, we estimate the energy and latency for few-shot learning with our proposed hardware. We implemented different structures (CNN, crossbar-based TLSH, and crossbar-based TCAM) in the memory augmented neural networks with specifically peripheral circuits, so their performance numbers are evaluated separately in the following sub-sessions. The evalutation is based on 65 nm CMOS technology node.

a) Memristive CNN controller

Using crossbar arrays to implement the convolutional neural networks (CNN) has been widely explored, so here we estimate the numbers based on the previously reported values[7,8]. The controller architecture and the computing macro are shown in Supplementary Fig. 21. Each computing macro consists of a bit line (BL) driver, a word line (WL) driver, 64×64 1T1R crossbars (4k devices), 64 samples, and hold (S&H) blocks, 16 4-to-1 muxes, 16 8-bit analog-to-digital converters (ADCs), and 16 shift-and-adds. Each real-valued input is divided into eight binary pulses with 10 ns pulse width and 0.2 V pulse amplitude. The memristors store the real-valued weights in their analog conductances. During each readout process, the output is first latched in the sample and hold circuit. Then every four analog output signals at the bit line will be converted to digital values with the shared ADC. This readout process will finish before the subsequent output signal arrives at the sample and hold.

For few-shot learning tasks with Omniglot dataset, our CNN controller has four convolutional layers and one fully-connected layer. A digital *ReLU* block is applied after each convolutional layer, and a digital *Max Pooling* block is used after every two convolutional layers. Our analysis considers two different cases. We first consider the channel-wise parallelism in the convolutional layer by unrolling convolutional kernels into crossbars. The second case considers both channel-wise and pixel-wise parallelism by duplicating convolutional kernels in the crossbar for parallel pixels. If only channel-wise parallelism is considered, an image with the size of 28×28 needs to be split into 784 vectors as shown in Supplementary Fig. 21. The detailed metrics of each module in the CNN controller are reported in the Supplementary Table 5. Most parameters for the IMA macro are taken from previous work with hardware demonstration on the CNN[8]. The parameters for the digital *ReLU* and *Max Pooling* are obtained based on previous work[7] and our own estimation. Based on the detailed metrics of each module in the CNN, we can estimate the performance of the crossbar-based CNN controller with channel-wise parallelism as shown in Supplementary Table 4, 5 and 6.

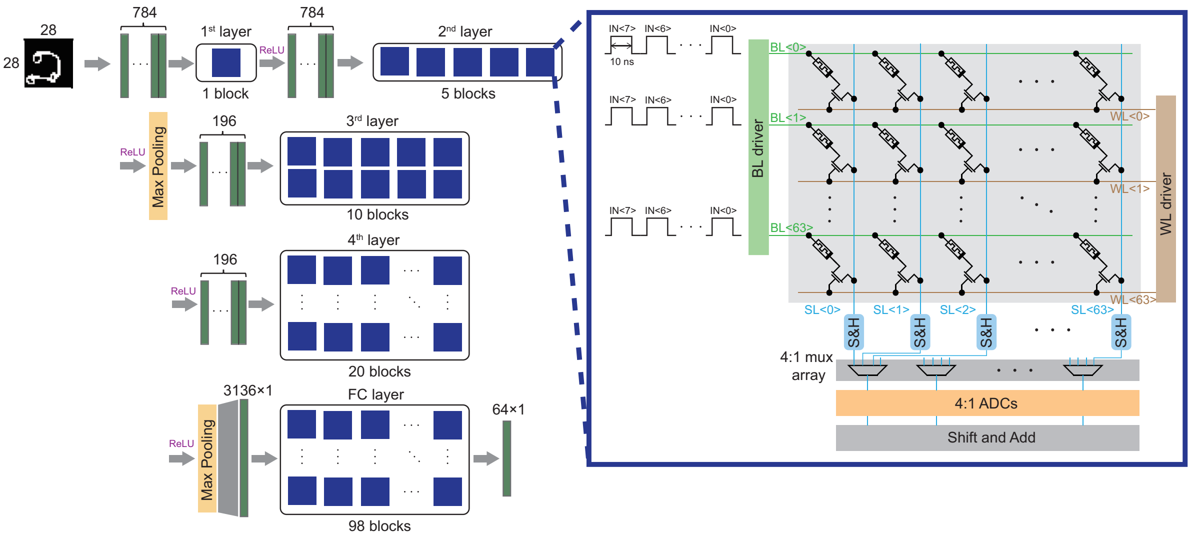

Supplementary Figure 21: CNN architecture and in-memory multiplication-and-add macro for Omniglot dataset. To realize the CNN in the memristive crossbars, the massive matrix multiplications are distributed in tiled 64×64 crossbar arrays. Each crossbar array contains a bit line (BL) driver, a word line (WL) driver, 4096 1T1R devices, 64 sample and hold (S&H) blocks, 16 4:1 muxes, 16 analog-to-digital converters (ADCs), and 16 shift-and-adds. Each real-valued input is divided into 8 binary input stream with 10 ns pulse width.

Supplementary Table 4: Detailed metrics of different components in CNN

Module	Area (μm^2)	Energy (nJ)	Num_blocks
1T1R array	37,118	59.0	134
BL driver	140,968	17.1	134
WL driver	140,968	17.1	134
S&H	670	5.6	134
4:1 mux array	11,152	82.0	134
ADC	3,216,000	2,785.3	134
Shift and add	467,526	700.2	134
ReLU	1200	1.1	4
MaxPooling	480	1.6	2
**Sum**	**4,016,442**	**3609.0**	-

Supplementary Table 5: Detailed metrics of each module in CNN controller with channel-wise parallelism for inference of one image from the Omniplot dataset

Layer	Ops	Energy on memristors (nJ)	Energy on circuits (nJ)	Latency (μs)	Num_blocks	Area (mm^2)
Conv2d #1	95,334	7.0	264.7	62.72	1	0.0300
Conv2d #2	32,062,464	16.2	1,323.4	62.72	5	0.1498
Conv2d #3	16,031,232	12.3	661.7	15.68	10	0.2996
Conv2d #4	32,087,552	23.1	1,323.4	15.68	20	0.5992
FC layer	802,815	0.5	33.1	0.08	98	2.9361
ReLU	-	-	1.1	4.00	4	0.0012
MaxPooling	-	-	1.6	4.00	2	0.0005
**Sum**	**81,937,407**	**59.0**	**3,609.0**	**164.88**	-	**4.0164**

Supplementary Table 6: Benchmark metrics of the crossbar-based CNN controller with channel-wise parallelism

Benchmark metric	Numbers
Performance	$81,937,407\text{ops}/164.88\,\mu\text{s} = 496.95\text{GOPS}$
Power	$(3609\,\text{nJ} + 59\,\text{nJ})/164.88\,\mu\text{s} = 22.25\,\text{mW}$
Energy efficiency	$496.95\text{GOPS}/22.25\,\text{mW} = 22.33\text{TOPS/W}$
Performance density	$496.95\text{GOPS}/4.0164\,\text{mm}^2 = 123.73\text{GOPS}/\text{mm}^2$

In the case where both channel-wise and pixel-wise parallelism are considered, multiple IMA blocks are configured for the same convolutional kernel to enable pixel-wise parallelism, and therefore more IMA blocks are needed. For example, a 28×28 image would require 784 duplicated blocks to process the 784 pixels at the same time. Based on the working principle and calculations above, we provide the same metrics for the CNN controller with both channel-wise and pixel-wise parallelism in Supplementary Table 7 and 8. The energy provided here is based on the future system with enough tiled arrays to store the entire CNN controller. In our experiment, due to a limited number of devices (24,576), the reprogramming of memristors will incur additional 1.69 μJ energy (consider each re-configuration requires 10 cycles of read-and-verify iterative programming on average). From the numbers, one finds that the pixel-wise parallelism significantly reduces the latency and throughput at expense of larger power and chip area.

Supplementary Table 7: Detailed metrics of each module in CNN controller with both channel-wise and pixel-wise parallelism

Layer	Ops	Energy on memristors (nJ)	Energy on circuits (nJ)	Latency (µs)	Num_blocks	Area (mm^2)
Conv2d #1	95,334	7.0	264.7	0.08	784	23.4900
Conv2d #2	32,062,464	16.2	1323.4	0.08	3,920	117.4400
Conv2d #3	16,031,232	12.3	661.7	0.08	1,960	58.7200
Conv3d #4	32,087,552	23.1	1323.4	0.08	3,920	117.4400
FC layer	802,815	0.5	33.1	0.08	98	2.9361
ReLU	-	-	1.1	4.00	4	0.0012
MaxPooling	-	-	1.6	4.00	2	0.0005
**Sum**	**81,937,407**	**59.0**	**3,609.0**	**8.40**	-	**320.0278**

Supplementary Table 8: Benchmark metrics of the crossbar-based CNN controller with both channel-wise and pixel-wise parallelism

Benchmark metric	Numbers
Performance	$81,937,407\text{ops}/8.40\,\mu\text{s} = 9.75\text{TOPS}$
Power	$(3609\,\text{nJ} + 59\,\text{nJ})/8.40\,\mu\text{s} = 436.67\,\text{mW}$
Energy efficiency	$9.75\text{TOPS}/436.67\,\text{mW} = 22.33\text{TOPS/W}$
Performance density	$9.75\text{TOPS}/320.0278\,\text{mm}^2 = 30.47\text{GOPS}/\text{mm}^2$

b) Memristor-based TLSH block

After the feature extraction using the crossbar-based CNN controller in the MANN, the ternary locality sensitive hashing (TLSH) is presented to convert the real-valued vectors into ternary hashing vectors. As we illustrated in the Supplementary Fig. 15**a**, the performance of the hashing barely degrades by the IR drop problem which exists in larger arrays. This is because, unlike the crossbar-based CNN where the wire resistances can change the weight value stored in the memristors, it won't affect the intrinsic stochasticities of the memristors in the array which is what TLSH requires. Therefore, in the TLSH macro design, we only need one 64×129 1T1R array for 128-dimensional ternary embeddings without tiling.

We present our TLSH macro with peripheral circuitry in Supplementary Fig. 22. Unlike the working principles of crossbar-based CNN, the TLSH functionality in crossbars needs to be fast but not as accurate as the CNNs since the output only contains '1', '0', and 'X'. Therefore, we

choose to directly encode the real-valued vectors into analog voltages using 8-bit digital-to-analog converters (DACs)[9]. We use the adjacent connection matrix to get the differential signals and the TLSH sensing block will obtain the ternary hashing codes. Detailed readout scheme and circuitry design can be found in Supplementary Note 2. With the provided macro design, we estimated about 0.846 ns latency for every hashing operation.

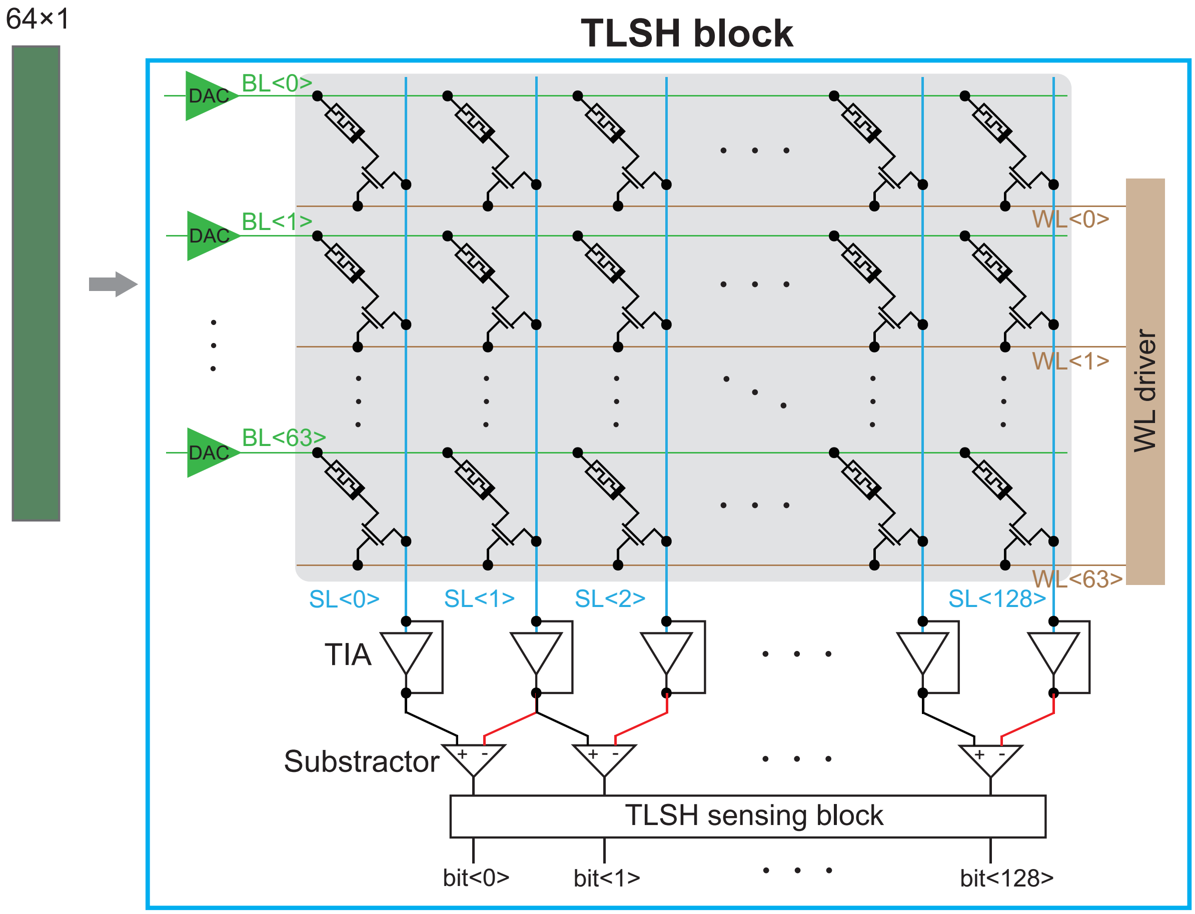

Supplementary Figure 22: CNN architecture and in-memory multiplication-and-add macro.

Based on the analysis, we report the detailed metric of TLSH macro and its performance in Supplementary Table 9 and 10. The metric of the array and DAC are from previous works[8,9]. The TIAs, Substractors, and TLSH sensing blocks are obtained by our in-house designers that simulated based on 180 nm PDK from a commercial foundry and the performance is forecasted by scaling the performance to 65 nm.

Supplementary Table 9: Detailed metrics of each module in the TLSH macro

Module	Area (μm^2)	Energy (pJ)	Size
1T1R array[8]	558	2.32	64×129
DAC[9]	480,000	224.00	64×1
TIA	913	3.53	1×129
Subtractor	675	3.49	1×128
TLSH sensing block	2,336	2.04	1×128
**Sum**	**484,482**	**235.38**	-

Supplementary Table 10: Benchmark metrics of the TLSH implemented in the crossbar array

Benchmark metric	Numbers
Performance	$(129 \times (64 \times 2 - 1))\text{ops}/0.846\,\text{ns} = 19.37\text{TOPS}$
Power	$235.38\,\text{pJ}/0.846\,\text{ns} = 278.23\,\text{mW}$
Energy efficiency	$19.37\text{TOPS}/278.23\,\text{mW} = 69.63\text{TOPS/W}$
Performance density	$19.37\text{TOPS}/0.484\,\text{mm}^2 = 39.98\text{TOPS}/\text{mm}^2$

c) TCAM searching

For TCAM, we consider a very large memory module 16 MB capacity (16× larger than the CNN without pixel-wise parallelism). Due to the IR drop problem, we divide the memory into $m \times n$ TCAM blocks as shown in Supplementary Fig. 23. Each TCAM block has 64×64 1T1R array. To store the 128-dimensional ternary vectors, we need 4 ($128 \times 2/64 = 4$) tiled arrays. Therefore in our design and simulations below, we use $m = 65,536/64 = 1024$ and $n = 4$.

We apply the threshold sensing technique in our TCAM design which is illustrated in Supplementary Note 3. Because of the tiled array, each output corresponding to the hamming distance between the query and one of the keys in the memory needs to be summed up by the output of the four blocks. Therefore, we apply one analog adder at every key which sums up the output voltage obtained from the TIA at each block. In addition, one comparator is used at every key to sense the output voltage. Once the output voltage is beyond the threshold voltage V_{thres}, the comparator will shut down all the transistors in that key. This will also let the sample and hold and ADC at that key remain idle, thus consuming zero energy. According to our simulation, after applying the threshold sensing, only 1/3 of the ADCs and S&Hs are activated during the search operation. The average sensing time on the crossbars per search is 5.2 ns. Based on the analysis above, we report the detailed metrics and benchmark for our TCAM module in the Supplementary Table 11 and 12.

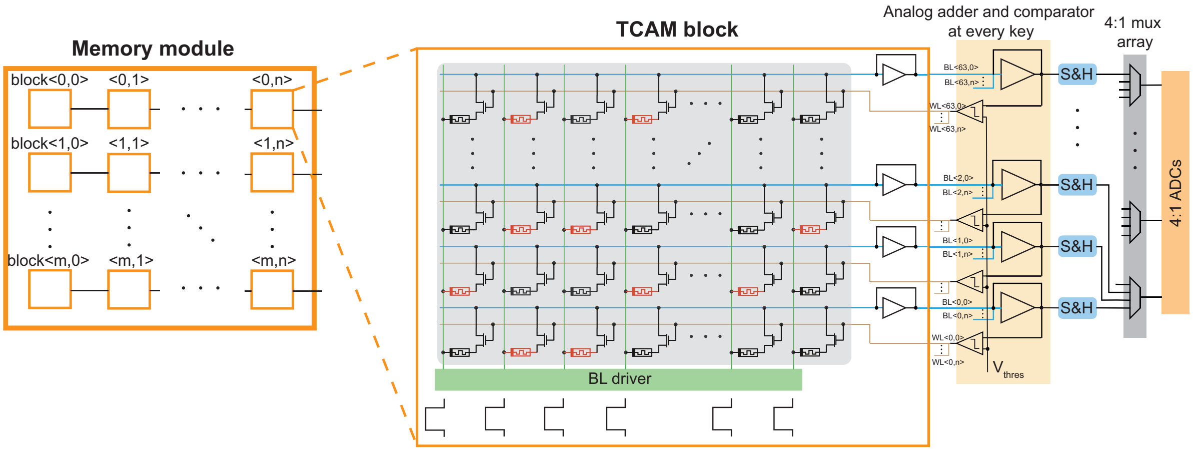

Supplementary Figure 23: CNN architecture and in-memory multiplication-and-add macro.

Supplementary Table 11: Detailed metrics of each module in the entire TCAM

Module	Area (μm^2)	Energy (pJ)	Num_blocks
Array	1,134,529	53,740	4,096
BL driver	4,308,992	819	4,096
S&H	5,120	22	1,024
mux array	90,112	328	1,024
ADC	2,457,6000	11,127	1,024
TIA	1,855,324	199,361	4,096
Analog adder	345,886	49,840	1,024
Comparator	330,957	49,840	1,024
**Sum**	**36,219,699**	**367,876**	-

Supplementary Table 12: Benchmark metrics of the crossbar-based TCAM

Benchmark metric	Numbers
Performance	$65,536 \times (2 \times 256 - 1)\text{ops}/10\,\text{ns} = 3,348.89\text{TOPS}$
Power	$367\,876\,\text{pJ}/10\,\text{ns} = 36.79\,\text{W}$
Energy efficiency	$3,348.89\text{TOPS}/36.79\,\text{W} = 91.03\text{TOPS/W}$
Performance density	$3,348.89\text{TOPS}/36.22\,\text{mm}^2 = 92.46\text{TOPS}/\text{mm}^2$

d) Summary

In summary, we estimated about 3.67 μJ per Omniglot image inference (5-way 1-shot) and 22.35

TOPS/W energy efficiency with our full crossbar-based MANN with 517× and 858× improvement over GPU (1.38 mJ and 0.026 TOPS/W on GPU). If we focus on the search operation on the memristors, the consumed energy for one search operation is only 0.82 pJ for the Omniglot dataset (128 bits with threshold sensing), 7,317× improvement over GPU. We further provide the energy efficiency and performance density comparison among the three different modules in the MANN which is shown in the Supplementary Table 13. We can find that with our design of the circuits, the TLSH and TCAM can achieve much higher energy efficiency and performance density than that of the conventional CNN. For the TLSH, the improvement mainly comes from the elimination of the ADCs in the peripheral circuits, offering a much higher speed. In terms of the TCAM, the improvements are mainly from the reduction of the ADCs' usage. With our threshold sensing technique, the circuit can adaptively float the wire where the corresponding hamming distance is not necessarily detected, saving the energy incurred by the large current and the corresponding dynamic power of the readout process.

Supplementary Table 13: Benchmark metrics of different modules in the MANN

Benchmark metric	CNN	TLSH	TCAM
Energy efficiency (TOPS/W)	22.33	69.63	91.03
Performance density (TOPS/mm^2)	0.03 ~ 0.12	39.98	92.46

For Mini-ImageNet dataset, we achieve 82.4 pJ per search (4,096 bits) on the crossbar arrays, over 5.1×10^4 improvement to GPU (4.2 µJ) on the same task[10]. The GPU latency numbers reported here are acquired using PyTorch Profiler[11], and the energy consumption numbers from the NVIDIA System Management Interface (nvidia-smi).

Those numbers can be further improved by choosing smaller input voltages and optimizing peripheral circuits with more advanced technology nodes. Here, we demonstrate that energy consumption can be improved by simply voltage scaling. In the experiment, we reduce the search voltage by a factor of 10: from 0.2 V to 0.02 V. In this case, the power consumption is lowered 100× with minor accuracy loss (from 76.8% to 77.5% for the 25-way 1-shot problem). It is noteworthy that the efficiency is not necessarily improving with larger crossbars[12]. We found the size of 64×64 or 128×128 is a sweet spot when considering the output current, matrix utilization rate, etc. Moreover, by using the sensing circuits described in Supplementary Note 3, we can achieve state-of-the-art energy efficiency over previous works. Quantitively, we compared search energy in the distance search operation with different approaches and listed them in the table below, clearly showing our approach's benefit and scalability. The result of the search energy is averaged over 11,875 search operations in the 25-way 1-shot task based on the experimentally measured data. To

make a fair comparison, we report the search energy per bit of our approach and achieve the lowest energy consumption over previous works.

Supplementary Table 14: Energy consumption per bit per search operation of TCAM

	PCM[13]	2FET[4]	Crossbar-TCAM	Crossbar-TCAM with threshold sensing	Crossbar-TCAM with voltage mode sensing
Energy consumption per bit per search (fJ)	2.500	0.400	0.175	0.064	0.011

Supplementary Note 5. Accuracy comparison with the state-of-the-art model

This note provides an accurate comparison between the proposed method in this work and a state-of-art method based on high-dimensional (HD) computing [13]. The comparison includes (1) baseline algorithms that are implemented in digital hardware, (2) the modified, hardware-friendly model that is implemented in digital hardware, and (3) the modified, hardware-friendly model that is implemented in an experimentally validated crossbar model. Specifically, in the reproduction of the high-dimensional method, we assume *pad=0* and *stride=1* for the convolutional layers.

(1) Accuracy with the proposed CNN and associative memory with cosine distance implemented in digital hardware, where numbers are represented as 32-bit floating-point. The result compares the high-dimensional approach using vectors with real-valued components. We also implemented the HD approach based on our understanding. The detailed comparison is reported below.

Supplementary Table 15: Accuracy with 512-dimensional, 32-bit floating point keys

Task	Ours	Our HD	Original HD[13]
5-way 1-shot	97.27%	97.74%	97.78%
20-way 5-shot	98.45%	98.09%	98.01%
100-way 5-shot	95.01%	94.62%	94.53%

(2) Accuracy with CNN, LSH, and TCAM implemented in the digital hardware. This result compares with the high-dimensional approach using vectors with bipolar components. The results are compared below. The dimension of the ternary or bipolar key vectors stored in the memory is 512 for all models.

Supplementary Table 16: Accuracy with 512-dimensional, ternary or bipolar keys without noise

Task	Ours	Our HD	Original HD[13]
5-way 1-shot	97.82%	97.53%	97.53%
20-way 5-shot	97.71%	98.07%	97.83%
100-way 5-shot	92.93%	93.62%	94.08%

(3) Accuracy with CNN, TLSH, and TCAM that simulated with our experimentally validated model, which takes into consideration the conductance relaxation, fluctuation, readout noise, wire resistance, *etc*. This result compares with the high-dimensional approach using vectors with binary

components that are simulated with their model based on phase-change devices (PCM). The results are compared below.

Supplementary Table 17: Accuracy with 512-dimensional, ternary or binary keys with non-idealities

Task	Ours	Original HD[13]
5-way 1-shot	97.64%	96.40%
20-way 5-shot	97.52%	97.60%
100-way 5-shot	91.56%	92.70%

All the results reported above are publicly available in an online repository [14].

4 Supplementary References

1. Li, J., Montoye, R. K., Ishii, M. & Chang, L. 1 mb 0.41 μm^2 2t-2r cell nonvolatile tcam with two-bit encoding and clocked self-referenced sensing. *IEEE Journal of Solid-State Circuits* **49**, 896–907 (2013).

2. Li, H. *et al.* Sapiens: A 64-kb rram-based non-volatile associative memory for one-shot learning and inference at the edge. *IEEE Transactions on Electron Devices* (2021).

3. Fedorov, V. V., Abusultan, M. & Khatri, S. P. An area-efficient ternary cam design using floating gate transistors. In *2014 IEEE 32nd International Conference on Computer Design (ICCD)*, 55–60 (IEEE, 2014).

4. Ni, K. *et al.* Ferroelectric ternary content-addressable memory for one-shot learning. *Nature Electronics* **2**, 521–529 (2019).

5. Laguna, A. F., Yin, X., Reis, D., Niemier, M. & Hu, X. S. Ferroelectric FET based in-memory computing for few-shot learning. *Proceedings of the ACM Great Lakes Symposium on VLSI, GLSVLSI* 373–378 (2019).

6. Razavi, B. The strongarm latch [a circuit for all seasons]. *IEEE Solid-State Circuits Magazine* **7**, 12–17 (2015).

7. Shafiee, A. *et al.* Isaac: A convolutional neural network accelerator with in-situ analog arithmetic in crossbars. *ACM SIGARCH Computer Architecture News* **44**, 14–26 (2016).

8. Yao, P. *et al.* Fully hardware-implemented memristor convolutional neural network. *Nature* **577**, 641–646 (2020).

9. Caragiulo, P., Mattia, O. E., Arbabian, A. & Murmann, B. A 2× time-interleaved 28-gs/s 8-bit 0.03-mm2 switched-capacitor dac in 16-nm finfet cmos. *IEEE Journal of Solid-State Circuits* **56**, 2335–2346 (2021).

10. Kazemi, A. *et al.* A flash-based multi-bit content-addressable memory with euclidean squared distance. In *2021 IEEE/ACM International Symposium on Low Power Electronics and Design (ISLPED)*, 1–6 (IEEE, 2021).

11. Paszke, A. *et al.* Pytorch: An imperative style, high-performance deep learning library. *Advances in neural information processing systems* **32** (2019).

12. Wan, W. *et al.* Edge ai without compromise: Efficient, versatile and accurate neurocomputing in resistive random-access memory. *arXiv preprint arXiv:2108.07879* (2021).

13. Karunaratne, G. *et al.* Robust high-dimensional memory-augmented neural networks. *Nature communications* **12**, 1–12 (2021).

14. Mao, R. ruibinmao/tlsh_mann: v1.0.0 (2022). URL `https://doi.org/10.5281/zenodo.7093390`.
